# Dual-ion Co-storage in a donor–acceptor covalent organic framework for high-performance low-temperature sodium-organic batteries

**DOI:** 10.1039/d6sc02423h

**Published:** 2026-05-14

**Authors:** Xinya Zhang, Shuangqin Yang, Yihao Zhang, Xiaoyan Zhang, Zaiwang Zhao, Yuan Chen

**Affiliations:** a College of Energy Materials and Chemistry, State Key Laboratory of New Textile Materials and Advanced Processing, Inner Mongolia University Hohhot 010070 China yuanchen@imu.edu.cn zwzhao@imu.edu.cn

## Abstract

The development of covalent organic framework (COF) cathodes for low-temperature sodium-ion batteries (SIBs) remains challenging due to sluggish reaction kinetics at low temperatures. Herein, we demonstrate a donor–acceptor (D–A) covalent organic framework constructed from p-type triphenylamine and n-type naphthalimide units as a robust cathode for low-temperature SIBs. The stable naphthalimide-based building blocks endow the constructed D–A framework with faster charge transport capability and enhanced redox kinetics, achieving a capacity of 135 mAh g^−1^ at 0.1 A g^−1^ and an impressive capacity retention of 80% after 2000 cycles at 1 A g^−1^. Remarkably, it maintains high capacities of 108 mAh g^−1^ at 0 °C and 85 mAh g^−1^ at −20 °C, with a capacity retention rate of 91% after 300 cycles at −20 °C. The dual-ion co-storage mechanism of Na^+^ and PF_6_^−^ has been elucidated through *in situ* spectroscopic characterization and theoretical calculations. This work provides a feasible molecular design strategy towards stable and high-performance porous organic cathodes for low-temperature SIBs.

## Abstract 2

A D–A type COF with a stable conjugated structure is reported for low temperature SIBs. This electrode enables efficient charge transfer, enhances redox kinetics, and exhibits good cycling stability under low-temperature conditions.

## Graphical abstract

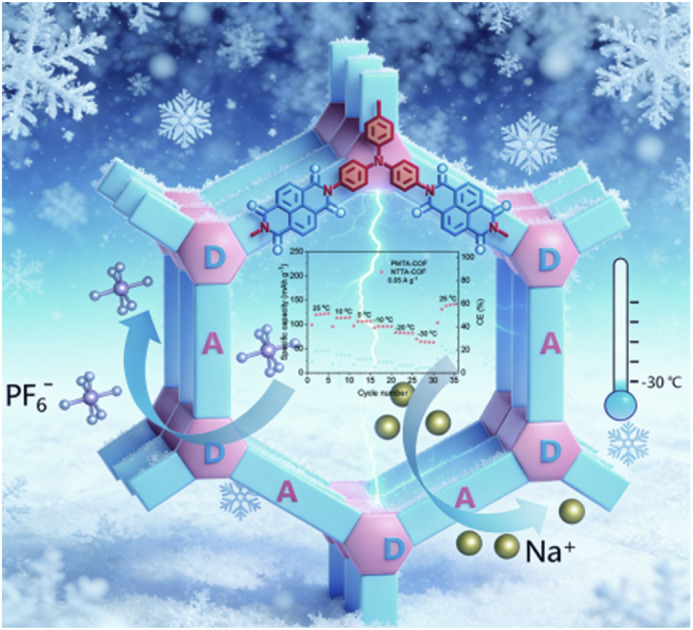

## Introduction

Sodium-ion batteries (SIBs) have garnered significant attention as promising candidates for next-generation large-scale energy storage systems, owing to the natural abundance of sodium resources, low cost, and physicochemical similarities to lithium-ion batteries (LIBs).^1–4^ However, the practical application of SIBs, especially under low-temperature conditions, remains challenging. Traditional inorganic electrode materials often suffer from severe structural degradation and sluggish ion diffusion kinetics during the repeated insertion/extraction of large Na^+^ ions,^5^ leading to rapid capacity fading and poor cycling stability at subzero temperatures.^6–8^ In contrast, organic electrode materials (OEMs) have emerged as attractive alternatives due to their structural diversity, environmental friendliness and sustainability.^9–14^ The flexible molecular structures of OEMs enable efficient accommodation of Na^+^ ions through coordination reactions, minimizing volume changes and providing inherent advantages for low-temperature applications.^11,15^ Furthermore, the structural tunability of organic materials enables the integration of diverse redox-active groups into framework polymers.^16,17^ This not only endows organic electrode materials with multiple active sites but also effectively addresses their dissolution challenges in electrolytes.^18^

Covalent organic frameworks (COFs), a class of crystalline porous polymers constructed from organic building blocks *via* robust covalent bonds, have recently gained considerable attention as organic electrode materials for rechargeable batteries.^19–23^ Their well-defined periodic structures, high specific surface areas, and tunable pore environments not only facilitate rapid ion transport but also provide abundant and accessible redox-active sites.^24^ More importantly, the inherent insolubility of COFs in organic electrolytes effectively mitigates the active material dissolution commonly encountered in small organic molecules, thereby ensuring enhanced cycling stability.^25^ Until now, the most commonly studied COF-based electrode materials often suffer from intrinsic low conductivity and relatively low operating voltage.^26^ The poor conductivity stems from the limited charge carrier mobility within the framework, which restricts electron transport and leads to sluggish redox kinetics.^27,28^ Meanwhile, the predominance of n-type redox-active sites (*e.g.*, carbonyls and imines) in most reported COF cathodes results in low working potentials,^29^ thereby limiting the energy density of the resulting batteries. To address these challenges, the design of donor–acceptor (D–A) structures is regarded as the most effective strategy.^30,31^ By integrating electron-rich donor units and electron-deficient acceptor units into the conjugated framework, D–A-type COFs can achieve narrowed HOMO–LUMO gaps and enhanced intramolecular charge transfer. The push–pull electronic effect between D and A units also promotes the delocalization of π-electrons, thereby enhancing carrier mobility and redox kinetics. Moreover, such bipolar D–A systems often exhibit dual-ion storage capability, where the donor and acceptor sites can reversibly host anions and cations,^32,33^ respectively, leading to increased specific capacity and higher operating voltage.^34^ Such rapid reaction kinetics may facilitate the efficient diffusion and storage of anions and cations under low-temperature conditions. Therefore, designing functional framework materials with D–A conjugated structures and systematically investigating their potential for application in low-temperature sodium-ion batteries is of significant scientific importance.

**Fig. 1:**
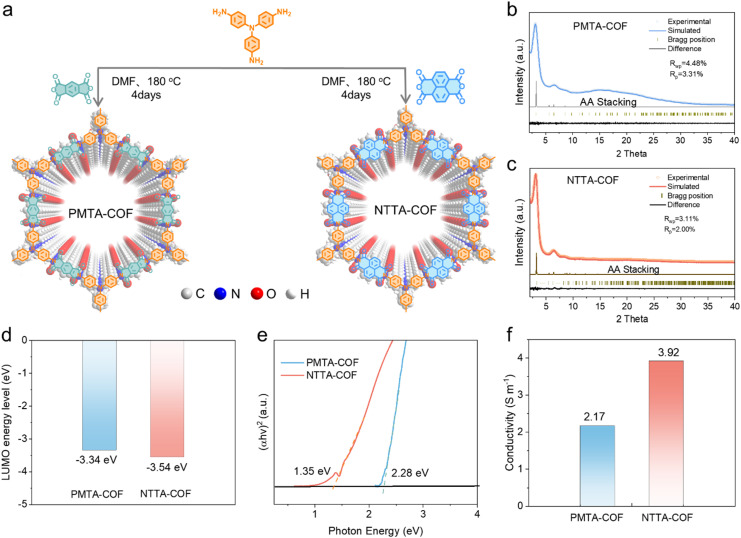
(a) Schematic diagram of the synthesis of PMTA-COF and NTTA-COF. Experimental and simulated XRD patterns of the (b) PMTA-COF and (c) NTTA-COF. (d) LUMO energy levels and (e) optical energy gaps *E*_g_ for PMTA-COF and NTTA-COF. (f) Electrical conductivities of two COF electrodes.

Herein, we synthesized two D–A-type COFs (NTTA-COF and PMTA-COF) by integrating redox-active triphenylamine electron-donor building blocks with n-type imide electron-acceptor units (Fig. 1a), and explored their application potential as cathodes for low-temperature SIBs. Owing to the more stable molecular structure and extended π-conjugated system of the naphthalimide unit, NTTA-COF exhibits a smaller gap compared to PMTA-COF. This effectively promotes charge transport and accelerates redox kinetics, thereby achieving higher capacity and cycling stability. Consequently, NTTA-COF exhibits a capacity of 135 mAh g^−1^ at 0.1 A g^−1^, maintaining an excellent capacity retention of 80% after 2000 cycles at 1 A g^−1^. Notably, it retains a high capacity of 108 mAh g^−1^ at 0 °C and 85 mAh g^−1^ at −20 °C, with a capacity retention rate of 91% after 300 cycles at −20 °C. The Na^+^/PF_6_^−^ co-storage mechanism was systematically investigated using *in situ*/*ex situ* spectroscopic techniques and density functional theory (DFT) calculations. This study offers a rational molecular design strategy for developing stable and high-performance porous organic electrodes for low temperature SIBs.

## Results and discussion

### Synthesis and characterization

Two bipolar D–A covalent organic framework materials, PMTA-COF and NTTA-COF (Fig. 1a, S1 and S2), were separately synthesized *via* an acylation reaction using acid anhydrides (PMDA or NTCDA) and tris(4-aminophenyl)amine (TAPA).^35–37^ The chemical bond information of PMTA-COF and NTTA-COF was investigated *via* Fourier transformed infrared spectroscopy (FT-IR). As shown in Fig. S3, the characteristic peak at around 1710 cm^−1^ is attributed to carbonyl stretching vibrations. The disappearance of the –NH_2_ peak at 3350 cm^−1^, accompanied by the observation of a new peak assigned to C–N–C linkages at 1350 cm^−1^ in the COFs, confirms the occurrence of imide cyclization reactions.^19^ Solid-state ^13^C-NMR spectroscopy reveals that the resonance signals of imine ring carbonyl carbon and C–N bond carbons in the two COFs are located at ∼163 ppm and ∼148 ppm, respectively; the overlapping peaks at 125–145 ppm are assigned to the phenyl carbons of the composite building blocks (Fig. S4).^36,38^

To elucidate the crystalline structure of COF powders, powder X-ray diffraction (PXRD) measurements were performed. The PXRD patterns of both NTTA-COF and PMTA-COF exhibit good crystallinity, with strong peaks at 3.0° and 3.1°, respectively, corresponding to reflections from the (110) plane (Fig. 1b and c). The experimental PXRD results of two COFs show excellent agreement with the simulated patterns of the AA-stacked model (Fig. S5, S6 and Table S1). The porous structures of the two COFs were evaluated *via* N_2_ adsorption–desorption measurements at 77 K. As shown in Fig. S7, NTTA-COF displays a permanent pore structure with a reversible Type I isotherm and a Brunauer–Emmett–Teller (BET) specific surface area of 634.4 m^2^ g^−1^. The pore size distribution curve reveals a pore size range of 1.5–3.0 nm, consistent with the AA-stacked model. The pore structure centered at 1.85 nm dominates in NTTA-COF due to the presence of smaller secondary pore structures. PMTA-COF exhibits a specific surface area of 429.8 m^2^ g^−1^, lower than that of NTTA-COF, which is attributed to its smaller pore size (∼1.67 nm). Scanning electron microscopy (SEM) images reveal that NTTA-COF has a nanoparticulate morphology (Fig. S8), and PMTA-COF displays a micron-sized bulk morphology (Fig. S9). Furthermore, high-resolution transmission electron microscopy (HRTEM) images (Fig. S10) indicate clear lattice spacings of 0.38 nm for NTTA-COF and 0.39 nm for PMTA-COF, corresponding to interlayer π–π stacking interactions. Thermogravimetric analysis (TGA) was employed to evaluate the thermal stability of both COFs under a nitrogen atmosphere (Fig. S11). NTTA-COF demonstrates excellent thermal stability, with only 6% weight loss below 491 °C, which is significantly better than that of most reported COF materials and polymer organic electrode materials (Table S2).

The lowest unoccupied molecular orbital (LUMO) energy levels for the two COFs are depicted in Fig. 1d. Compared to PMTA-COF (−3.34 eV), NTTA-COF displays a lower LUMO level of −3.54 eV, suggesting a stronger electron affinity that contributes to an elevated reduction potential (Fig. S12). Due to the stable π-conjugated framework and ordered covalent crystalline structure, NTTA-COF achieves a low optical energy gap (*E*_g_) of 1.35 eV (Fig. 1e) compared to that of PMTA-COF (2.28 eV), resulting in high intrinsic conductivity that facilitates redox reactions with low energy barriers. For conductivity measurements, NTTA-COF or PMTA-COF was mixed with conductive carbon and polytetrafluoroethylene in a mass ratio of 6 : 3 : 1. The extended π-conjugation endows the NTTA-COF electrode with efficient electron delocalization pathways, leading to a superior electrical conductivity of 3.92 S m^−1^ compared to that of the PMTA-COF electrode (2.17 S m^−1^) (Fig. 1f and S13). Such enhanced conductivity facilitates rapid electron transfer, thereby promoting highly kinetic and reversible redox reactions. Furthermore, the extended π-conjugated aromaticity of NTTA-COF is visually revealed by the localized orbital locator function (ELF) map (Fig. S14), demonstrating prominent electron delocalization pathways throughout its framework.

### Electrochemical performance

**Fig. 2:**
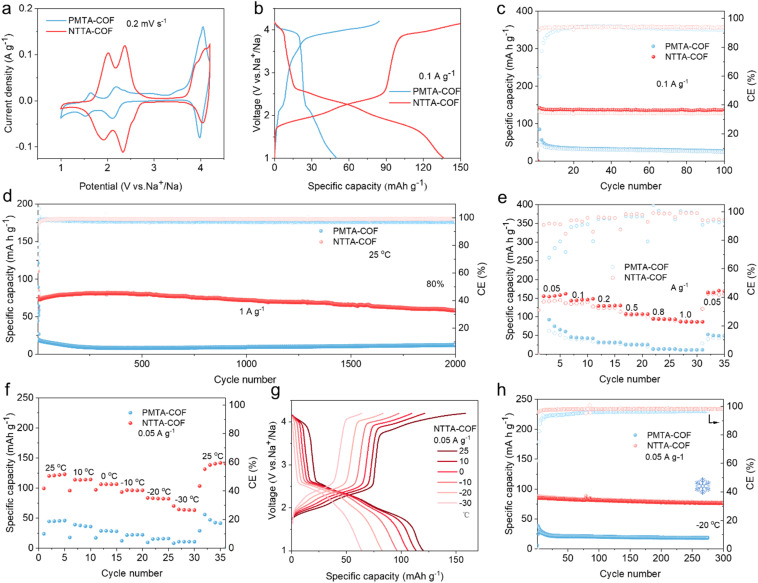
(a) CV curves of PMTA-COF and NTTA-COF at a scan rate of 0.2 mV s^−1^. (b) The galvanostatic charge–discharge curves of PMTA-COF and NTTA-COF at 0.1 A g^−1^. Cycling performance of PMTA-COF and NTTA-COF at (c) 0.1 and (d) 1 A g^−1^. (e) Rate performance of PMTA-COF and NTTA-COF. (f) Temperature-dependent galvanostatic cycling and (g) the corresponding charge–discharge curves at 0.05 A g^−1^ of PMTA-COF and NTTA-COF. (h) Cycling performance of PMTA-COF and NTTA-COF at 0.05 A g^−1^ and −20 °C.

The electrochemical performance of NTTA-COF and PMTA-COF was systematically evaluated using coin-type half-cells with sodium metal as the counter electrode. Cyclic voltammetry (CV) tests reveal three distinct pairs of redox peaks for both COF electrodes (Fig. 2a and S15). The redox peaks of NTTA-COF are located at 2.0/1.87 V, 2.34/2.3 V, and 4.08/4.02 V, while the corresponding peaks of PMTA-COF appear at 1.63/1.53 V, 2.18/2.11 V, and 4.04/3.96 V. The redox peaks below 2.5 V originate from reversible n-type reactions in the imide units, accompanied by Na^+^ insertion/extraction, while the peaks above 3.8 V correspond to p-type redox reactions between the triphenylamine units and PF_6_^−^ anions.^30,39^ Regarding the additional small redox peaks observed in the CV curves of NTTA-COF (*e.g.*, the oxidation peak at about 3.8 V and the reduction peak at about 2.6 V), this phenomenon is attributed to charge trapping during discharge.^40^ The high energy barrier required to dissociate trapped charges leads to significant polarization during their release. Thus, the oxidation peak near 3.8 V originates from the release of confined n-type charges, a characteristic feature commonly observed in bipolar electrode materials.^41–44^ Notably, NTTA-COF exhibits higher redox potentials, primarily attributed to its lower LUMO energy level (Fig. 1d). Simultaneously, the CV curve of NTTA-COF shows significantly larger integrated areas, indicating superior charge storage capability. This characteristic directly relates to its larger specific surface area, which provides favorable conditions for exposing more redox-active sites, thereby significantly enhancing storage capacity. Galvanostatic charge–discharge curves further confirm the excellent electrochemical performance of NTTA-COF (Fig. 2b and S16). NTTA-COF exhibits a reversible capacity of 135 mAh g^−1^ at 0.1 A g^−1^, with no capacity decay even after 100 cycles, significantly outperforming PMTA-COF (Fig. 2c). The differential capacity curves (d*Q*/d*V*) show strong consistency with both CV and charge–discharge profiles (Fig. S17), demonstrating good electrochemical reversibility of NTTA-COF. In terms of long-term cycling stability, NTTA-COF demonstrates outstanding performance (Fig. 2d and Table S3). After 2000 cycles at 1.0 A g^−1^, the capacity loss per cycle is only 0.013% (80% capacity retention, Fig. 2d), with average coulombic efficiency maintained above 99%, and the specific capacity consistently surpasses that of PMTA-COF. Furthermore, NTTA-COF also exhibits significantly better rate capability (Fig. 2e and S18). At current densities of 0.05, 0.1, 0.2, 0.3, 0.8, and 1.0 A g^−1^, the specific capacities of NTTA-COF reach 148, 136, 124, 106, 94, and 87 mAh g^−1^, respectively. When the current density returns to 0.05 A g^−1^, the specific capacity recovers to 150 mAh g^−1^, demonstrating excellent structural stability and electrochemical reversibility. Differential capacity curves further confirm the stability of redox peak positions at different current densities (Fig. S19). Moreover, the rate performance of NTTA-COF also surpasses that of some reported ambipolar organic electrode materials, further confirming its excellent kinetic properties (Fig. S20 and Table S4). These findings demonstrate that incorporating stable molecular building blocks into the structural design of D–A-type COFs effectively enhances the electrochemical performance of organic cathode materials.

Low-temperature electrochemical performance was further investigated. Compared to the room-temperature profiles, both NTTA-COF and PMTA-COF retain similar redox features, with NTTA-COF exhibiting superior electrochemical reversibility (Fig. S21). In addition, NTTA-COF demonstrates good electrochemical tolerance within the temperature range of 25 °C to −30 °C (Fig. 2f and g). At 0.05 A g^−1^, when the temperature decreases from 25 to 10, 0, −10, −20, and −30 °C, the discharge capacities remain at 118.4, 108.1, 97.3, 85.2, and 64.3 mAh g^−1^, corresponding to 80%, 73%, 66%, 58% and 43% of its room temperature capacity, respectively. When the temperature increases directly from −30 to 25 °C, the capacity recovers to 148 mAh g^−1^, indicating excellent structural reversibility under low-temperature conditions. In contrast, PMTA-COF shows poor low-temperature adaptability (Fig. 2h, S22 and S23a), with a capacity of only 15.3 mAh g^−1^ at −20 °C, representing just 34.7% of its room-temperature capacity. Additionally, NTTA-COF also exhibits excellent long-term cycling stability at −20 °C and 0.05 A g^−1^. After 300 cycles, the capacity retention remains as high as 91% (Fig. 2h). The minimal polarization and well preserved voltage plateaus observed at different cycle numbers further confirm the stable framework structure (Fig. S23b). Compared to some inorganic materials, NTTA-COF exhibits advantages in terms of capacity and stability (Table S5). In addition, we also tested the low-temperature electrochemical performance of NVP. As illustrated in Fig. S24, NVP delivers a specific capacity of merely 60 mAh g^−1^ at −20 °C, accompanied by an increase in voltage polarization. In contrast, NTTA-COF exhibits a significantly higher capacity than NVP at the same temperature, with no perceptible polarization observed. These results demonstrate that NTTA-COF possesses superior low-temperature kinetics compared to conventional inorganic electrode materials.

We further assembled an NTTA-COF//HC full cell using NTTA-COF as the cathode and hard carbon (HC) as the anode. As shown in Fig. S25, the full cell delivers a specific capacity of 115 mAh g^−1^ at a current density of 0.1 A g^−1^ at room temperature. Owing to the low potential of the HC anode, the NTTA-COF//HC full cell also exhibits a high average discharge voltage. The energy density of the full cell reaches 264.5 Wh kg^−1^ based on the mass of the cathode active material, and 176.3 Wh kg^−1^ when calculated based on the total mass of active materials from both electrodes, demonstrating promising energy output characteristics. Notably, the full cell also demonstrates excellent low-temperature performance, delivering a specific capacity of 75 mAh g^−1^ at −20 °C while maintaining good cycling stability.

**Fig. 3:**
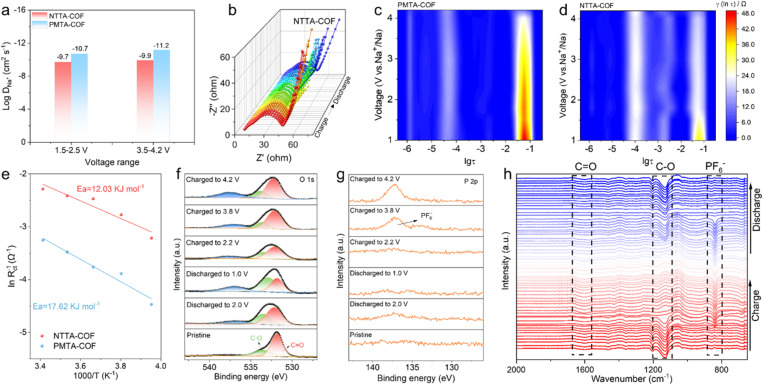
(a) The average Na-ion diffusion coefficients for PMTA-COF and NTTA-COF electrodes in different voltage ranges. (b) *In situ* Nyquist plots collected at various charged and discharged states of NTTA-COF. *In situ* DRT profiles for (c) PMTA-COF and (d) NTTA-COF. (e) The activation energies of PMTA-COF and NTTA-COF were deduced from the Arrhenius curve. High-resolution (f) O 1s and (g) P 2p XPS spectra of NTTA-COF. (h) *In situ* ATR-IR spectra of NTTA-COF.

The CV curves at different scan rates (0.2–5.0 mV s^−1^) were employed to investigate the reaction kinetics differences between NTTA-COF and PMTA-COF. As shown in Fig. S26, two types of redox peaks indicate that the two COF electrodes exhibit dual-ion storage behavior during electrochemical reactions. Obviously, two distinct pairs of redox peaks were observed between 1.5 and 2.5 V in NTTA-COF, with a third pair appearing near 3.9 V. The calculated *b*-values (*b*_1_ = 0.8, *b*_2_ = 0.93, *b*_3_ = 0.92, *b*_4_ = 0.61, *b*_5_ = 0.93, and *b*_6_ = 0.86) indicate that the capacitive process dominated in NTTA-COF. In contrast, the redox peaks for PMTA-COF become less pronounced with increasing scan rate, and the corresponding calculated *b*-values are significantly lower than those for NTTA-COF, suggesting lower redox kinetics for the PMTA-COF electrode. In addition, the contribution rate of the pseudocapacitance was also calculated, and the contribution of the pseudocapacitance also increased as the scanning speed increased. The pseudocapacitive contribution of NTTA-COF increases from 62% to 90%, which is significantly higher than that of PMTA-COF (Fig. S26). These results indicate that the charge storage mechanism is predominantly governed by fast interfacial pseudocapacitive behavior, which endows NTTA-COF with exceptional electrochemical kinetics. The galvanostatic intermittent titration technique (GITT) was also applied to analyze the ion diffusion coefficients (*D*) of the two electrodes (Fig. S27). The calculated results show that the log D values of NTTA-COF remain stable between −9.0 and −10.0 cm^2^ s^−1^ during both charging and discharging processes, outperforming those of PMDA-COF. Notably, NTTA-COF exhibits superior reaction kinetics regardless of whether it stores cations or anions (Fig. 3a), with ion diffusion rates unaffected by ion type. Furthermore, electrochemical impedance spectroscopy (EIS) was employed to investigate reaction kinetics, further elucidating the underlying mechanism for the enhanced capacity of NTTA-COF compared to PMTA-COF. As shown in Fig. S28 and S29, NTTA-COF consistently maintains significantly lower impedance than PMTA-COF at different cycle numbers, confirming the excellent electrode reaction kinetics and stability. In addition, *in situ* monitoring of EIS at different redox states further elucidated the kinetic differences between PMTA-COF and NTTA-COF electrodes. As shown in Fig. S30 and S31, the reaction resistance of the PMTA-COF electrode decreases significantly during charging and recovers after discharging. Although the NTTA-COF electrode exhibits a similar trend, resistance variation is minimal, and the reaction resistance remains significantly lower than that of the PMTA-COF electrode throughout the entire cycling period (Fig. 3b, S31 and Table S6). After converting the EIS data from the charging process into a distribution of relaxation times (DRT) function (Fig. 3c, d and S32), the NTTA-COF electrode shows lower peak intensity and a more gradual change in reaction resistance, indicating superior reaction kinetics. These findings demonstrate that D–A-type NTTA-COF electrodes with structurally stable molecular configurations exhibit enhanced charge transfer capability, enabling superior kinetic performance under low-temperature conditions (Fig. S33). Specifically, when the temperature decreases from room temperature (25 °C) to −20 °C, the charge transfer resistance of NTTA-COF electrodes increases only from 9.82 Ω to 24.86 Ω, showing minimal variation and strong low-temperature adaptability. In contrast, the PMTA-COF electrode consistently exhibits significantly higher impedance values than the NTTA-COF electrode over the entire low-temperature range (−20 °C to 25 °C), with more pronounced fluctuations in resistance as temperature changes. When the temperature drops to −20 °C, the impedance increases to 87.3 Ω, representing an increase of 61.45 Ω compared to room temperature. The activation energies (*E*_a_) for interfacial charge transfer at the two COF cathodes were calculated by fitting electrochemical impedance spectra (Fig. 3e). Based on the Arrhenius equation, the *E*_a_ value for alternating storage of Na^+^/PF_6_^−^ in NTTA-COF is 0.125 eV, lower than the 0.183 eV observed in PMTA-COF. This result indicates that the D–A-type COF framework with a stable π-conjugated structure can activate high-kinetic interfacial charge transfer and redox reactions, efficiently triggering redox-active sites in the NTTA-COF cathode through a lower energy barrier, thereby maintaining efficient charge transport at low temperatures.

### Electrochemical mechanism

To elucidate the dual-ion storage mechanism of the NTTA-COF electrode, the interaction mechanism between NTTA-COF and the charge carriers was provided by XPS analysis. During the discharge process, the intensity of the C=O peak significantly decreases while the C–O peak intensity correspondingly increases (Fig. 3f). Conversely, the exact opposite trend is observed during charging, suggesting that the carbonyl groups are potential n-type active sites. In addition, the evolution in the P 2p spectrum exhibits obvious changes only during charging, with the signal intensity of the P–F peak at 4.2 V being significantly higher than at 2.2 V and 1 V (Fig. 3g). Concurrently, a distinct = N^+^- characteristic peak at 401.8 eV emerges in the N 1s spectrum during charging (Fig. S34). These characteristic signals completely disappear during the discharge process, indicating that the triphenylamine-based p-type active sites undergo oxidation and bind PF_6_^−^ during charging. Furthermore, the FT-IR spectroscopy measurement was performed on the electrodes under different charge–discharge states. As shown in Fig. S35, the characteristic peak of C=O at 1680 cm^−1^ gradually decreases during the discharge process, confirming that the carbonyl groups serve as the primary n-type active sites for Na^+^ coordination.^45^ When charged to 3.6 V, the C=O peak intensity almost completely recovers to its initial state, demonstrating the reversible sodiation of C=O. Upon further charging to 4.2 V, the characteristic peak at 855 cm^−1^ assigned to PF_6_^−^ anions shows significant enhancement, indicating successful anion insertion into the framework. The systematic evolution of both C=O/C–O and PF_6_^−^ characteristic peaks in the *in situ* FTIR spectra provides compelling evidence for the consecutive storage mechanism of both Na^+^ and PF_6_^−^ in NTTA-COF (Fig. 3h). The XPS analysis results corroborate FT-IR, collectively confirming the dual-ion storage mechanism of NTTA-COF, wherein Na^+^ is stored during discharge and PF_6_^−^ is incorporated during charging.

### Theoretical calculations

**Fig. 4:**
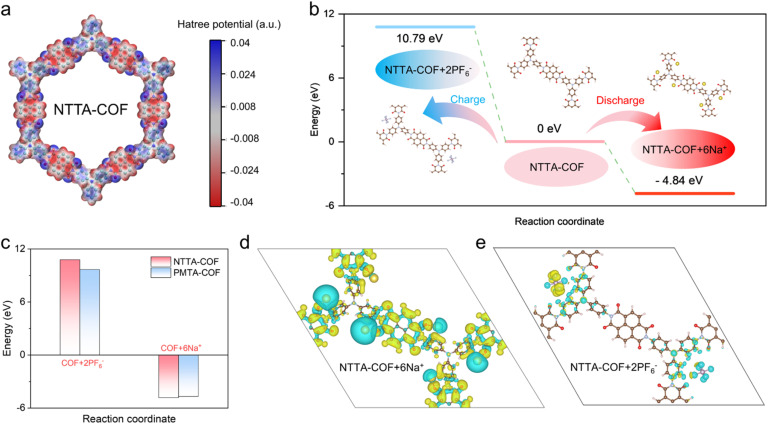
(a) Electrostatic potential diagram of NTTA-COF. (b) Calculated energies of Na-ions during the possible redox routes of NTTA-COF. (c) Calculated energy comparison of Na-ions during the possible redox routes of NTTA-COF and PMTA-COF. Charge density difference of (d) NTTA-COF + 6Na^+^ and (e) NTTA-COF + 2PF_6_^−^. The turquoise and yellow regions correspond to charge depletion and charge accumulation, respectively.

The dual-ion storage mechanism of NTTA-COF cathodes was further elucidated through density functional theory (DFT) calculations. Electrostatic potential analysis identifies electron-rich regions surrounding oxygen atoms in both COFs, suggesting preferential Na^+^ coordination sites (Fig. 4a and S36). Additionally, the triphenylamine center exhibits nearly neutral molecular electrostatic potential values, indicating their potential to accommodate anions (PF_6_^−^) in oxidized states. Based on these results, the structural evolution of NTTA-COF during electrochemical processes was established (Fig. 4b). During charging (2.8 V to 4.2 V), PF_6_^−^ anions bind to the triphenyl nitrogen active sites, forming an anion doped product (NTTA-COF + 2PF_6_^−^) with a free energy of 10.79 eV. During the discharge process (2.8 V to 1.0 V), Na^+^ cations coordinate with the carbonyl active sites in the naphthalimide units, generating a cation-doped product (NTTA-COF + 6Na^+^) with a free energy of −4.84 eV. The energy of Na^+^-containing NTTA-COF is lower than that of PMTA-COF (Fig. 4c and S37), indicating that Na^+^ tends to bind more favorably with NTTA-COF, forming a thermodynamically more stable structure.

The charge density difference iso-surfaces were simulated to reveal the bonding properties between Na^+^ and the carbonyl group, as well as between the PF_6_^−^ anion and the N-site of triphenylamine (Fig. 4d, e and S38). As shown in Fig. 4d, upon Na^+^ binding to NTTA-COF, charge depletion around Na^+^ and charge accumulation in the carbonyl region are clearly observed, indicating a significant charge transfer tendency toward the electroactive C=O site and the formation of a strong chemical bond between them. When NTTA-COF stores PF_6_^−^ anions, it exhibits charge distribution patterns opposite to those observed during Na^+^ storage (Fig. 4e).

## Conclusions

In summary, a stable D–A type COF cathode was designed and synthesized for low-temperature sodium-ion batteries. Due to the stable conjugated structure and narrower bandgap, NTTA-COF exhibits excellent electrochemical performance compared to its counterpart (PMTA-COF). As a result, the NTTA-COF electrode achieves a reversible capacity of 135 mAh g^−1^ at 100 mA g^−1^ and 25 °C. Even at a high rate of 1 A g^−1^, the capacity remains at 87 mAh g^−1^ and maintains stable cycling performance for 2000 cycles with a capacity retention rate of 80%. Regarding low-temperature performance, NTTA-COF also delivers a capacity of 108 mAh g^−1^ at 0.05 A g^−1^ and 0 °C, representing 80% of its room-temperature capacity. Remarkably, it retains a high reversible capacity of 85 mAh g^−1^ even at −20 °C, with a capacity retention rate of 91% after 300 cycles. A series of characterization methods and theoretical calculations demonstrate the reversible insertion/extraction behavior of PF_6_^−^ and Na^+^ at the redox active sites of the COF during charge/discharge cycles. This study provides a viable strategy to design D–A porous organic cathode materials with high stability for low-temperature sodium-ion batteries.

## Author contributions

X. Zhang carried out the experiments, analyzed the data, and wrote the original manuscript. S. Yang and Y. Zhang analyzed the data and participated in the discussion. X. Zhang guided writing and edited the manuscript. Y. Chen and Z. Zhao supervised the whole project, guided writing, and reviewed, and edited the manuscript. Y. Chen guided writing, and reviewed, and edited the manuscript.

## Conflicts of interest

The authors declare no conflicts of interest.

## Supplementary Material

SC-017-D6SC02423H-s001

## Data Availability

Reasonable requests for additional information can be made to the corresponding authors. Supplementary information (SI): detailed experimental methods, synthesis, structural characterization and additional experimental data supporting this article have been provided in the SI. See DOI: https://doi.org/10.1039/d6sc02423h.
