# Supplementary material for: Dual-ion Co-storage in a donor–acceptor covalent organic framework for high-performance low-temperature sodium-organic batteries
Source: Chem Sci. 2026 May 14;17(26):12981–9. doi: 10.1039/d6sc02423h (PMC13195449; doi:10.1039/d6sc02423h)
Supplement: SC-017-D6SC02423H-s001 [file SC-017-D6SC02423H-s001.pdf]

Supplementary Information (SI) for Chemical Science.
This journal is © The Royal Society of Chemistry 2026

Dual-Ion Co-storage in a Donor-Acceptor Covalent Organic Framework for High-Performance Low-Temperature Sodium-Organic Batteries

Xinya Zhang[+], Shuangqin Yang[+], Yihao Zhang[+], Xiaoyan Zhang, Zaiwang Zhao,* and Yuan Chen*

X. Zhang, S. Yang, Y. Zhang, X. Zhang, Prof. Dr. Z. Zhao, Prof. Dr. Y. Chen
College of Energy Materials and Chemistry
State Key Laboratory of New Textile Materials and Advanced Processing
Inner Mongolia University
Hohhot, 010070, China
E-mail: yuanchen@imu.edu.cn; zwzhao@imu.edu.cn

[+]These authors contributed equally to this work.

Experimental Section

Materials. Acetone, ethanol, N-methylpyrrolidone (NMP), N,N-dimethylformamide (DMF) were purchased from Energy Chemical. Polyvinylidene fluoride (PVDF), 1M $NaPF_6$/DIGLYME were purchased from DoDoChem. Tris(4-aminophenyl)amine (TAPA), 1,4,5,8-naphthalene tetrakis anhydride (NTCDA) and homophthalic tetrakis anhydride (PMDA) were purchased from Shanghai Titan Technology Co., Ltd. All purchased reagents and solvents were used without further purification.

Synthesis of PMTA-COF.[1] A mixture of TAPA (63 mg, 0.15 mmol), PMDA (49 mg, 0.225 mmol), and anhydrous DMF (5.0 mL) was added to a 250 mm × 10 mm Pyrex tube. Subsequently, the Pyrex tube was sonicated for 30 minutes and then degassed by three freeze-extraction-thaw cycles. Subsequently, the flame-sealed tubes were placed in an oven set at 180°C for three days. The solid was obtained by filtration and washed three times consecutively with DMF, acetone, and ethanol, respectively. The target product was then subjected to vacuum drying at a temperature of 80°C for a duration of 12 hours, resulting in the formation of a brownish-red powder. Yield: 76.6 mg (80%).

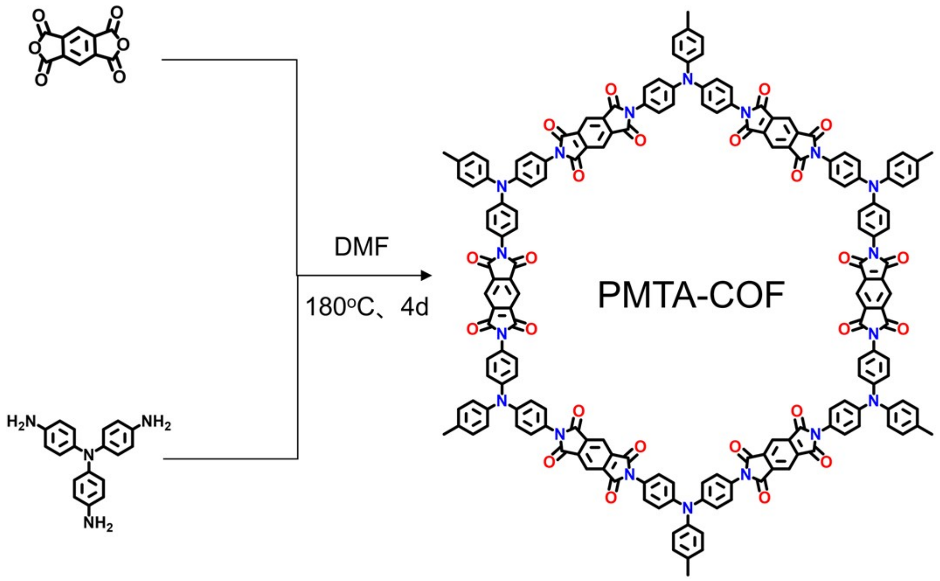

Figure S1. Synthesis of PMTA-COF.

Synthesis of NTTA-COF.[2] A mixture of TAPA (63 mg, 0.15 mmol), NTCDA (60 mg, 0.225 mmol), and anhydrous DMF (5.0 mL) was added to a 250 mm × 10 mm Pyrex tube. Subsequently, the Pyrex tube was sonicated for 30 minutes and then degassed by three freeze-extract-thaw cycles. Subsequently, the flame-sealed tubes were placed in an oven set at 180°C for three days. The solid was obtained by filtration and washed three times consecutively with

DMF, acetone, and ethanol, respectively. The target product was then subjected to vacuum drying at a temperature of 80℃ for a period of 12 hours, which resulted in the formation of a brown powder. Yield: 85.3 mg (89%).

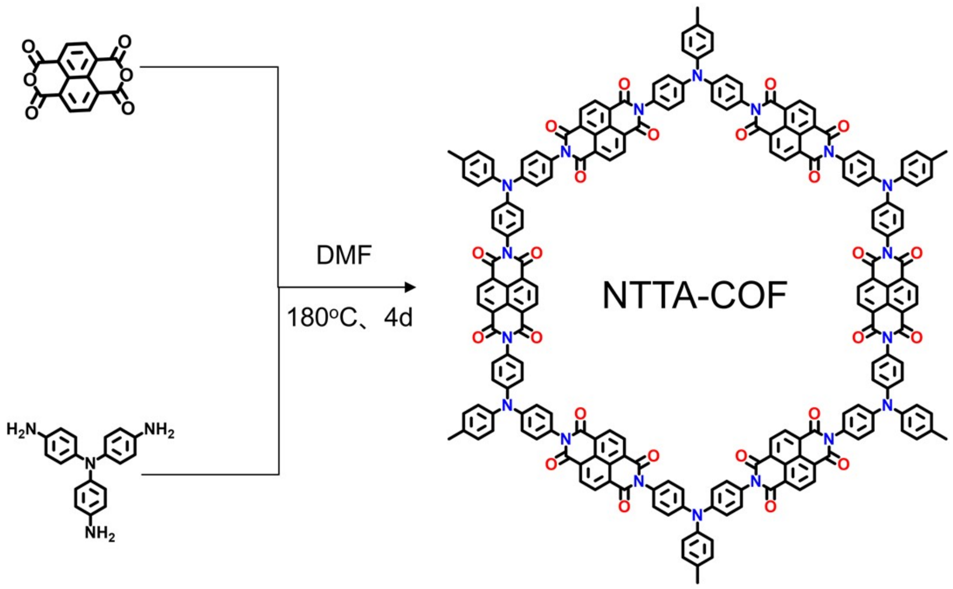

Figure S2. Synthesis of NTTA-COF.

Characterizations

Fourier Transform Infrared Spectroscopy (FTIR) spectra were recorded on RAffinity-1s from 4000 cm^{-1} to 400 cm^{-1}. In-situ FTIR was performed with a Nicolet IS50 (Thermo fisher) spectrometer instrument. In situ ATR-FTIR cell was purchased from the Beijing Scistar Technology Co. Ltd. Thermogravimetric analysis (TGA) was carried out on a Perkin-Elmer instrument in the temperature range 40 ~ 800°C under nitrogen atmosphere with a rate of 10°C min^{-1}. Powder X-ray diffraction (PXRD) was measured at room temperature on Bruker D8 Advance diffractometer with Cu Kα radiation (λ= 1.541 Å) operating at 40 kV and 40 mA. X-ray photoelectron spectroscopy (XPS) data were performed on an ESCALAB 250Xi system with a monochromatic Al Kα X-ray source (Thermo VG Scientific) to study the surface element changes. All spectra were charge corrected with respect to the C 1s component with a binding energy of 284.8 eV and analyzed using Thermo Avantage software. The authors would like to thank Xiao Ming Li from SCI-GO (https://www.sci-go.com) for the XPS analysis. Nitrogen adsorption and desorption tests were performed on an ASAP 2020 PLUS HD88 at 77 K. All samples were degassed at 120 °C for 5 hours before testing. Scanning electron microscope (SEM) images were recorded on Zeiss field-emission scanning electron microscope. Transmission Electron Microscopy (TEM) images were acquired at Shiyanjia Lab (https://www.Shiyanjia.com) using a Hitachi HT7700 instrument operated at an acceleration

voltage of 200 kV. Solid-state ^{13}C NMR spectra obtained on a 600 MHz Bruker Advance spectrometer.

Electrochemical measurements

The electrodes were fabricated from a mixture of active material, conductive carbon, and PVDF binder, with a mass ratio of 6:3:1. The mixture was dispersed in water, thoroughly ground, and subsequently cast on aluminum foil. The mixture was then dried in a vacuum oven at 80°C for 12 hours. The electrodes were cut into 14-mm disks, and the mass loading of the active substance in the electrode was approximately 1.2 mg cm^{-2}. The following materials were utilized: 1 M $NaPF_6$ in DIGLYME, glass fiber, and sodium foil, which were used as electrolyte, separator, and counter electrode, respectively. The CR2032 coin batteries were assembled in an argon-filled glovebox, where the levels of water and oxygen were maintained below 0.1 parts per million (ppm). Electrochemical measurements were employed on a LAND 2001A apparatus (Wuhan, China) within the voltage range of 1.0 to 4.2 V (vs. Na/Na^+). Cyclic voltammetry (CV) and electrochemical impedance spectroscopy (EIS) were performed on a Biologic electrochemical workstation within a scanning rate of 0.5 mV s^{-1} and a frequency range of 10^6 ~ 10^{-1} Hz , respectively. High and low temperature test chamber (ADX-GDW-100L) was used for temperature control. Prior to the initiation of the low-temperature electrochemical test, the battery was maintained at a low temperature for a duration of two hours. Galvanostatic intermittent titration technique (GITT) tests were carried out using LANHE-CT2001A system (Wuhan, China) in the potential ranging from 1.0 to 4.2 V (vs. Na^+/Na). GITT was tested at 0.1 A g^{-1} with a constant current pulse of 5 min and a relax of 30 min. For full SIBs, NTTA-COF was used as cathode, hard carbon (HC) as the anode and 1M $NaPF_6$/DIGLYME was applied as the electrolyte. The active mass ratio between cathode and anode is 1:0.5. The specific capacity of full cell was calculated based on the mass of the active material in the cathode. Before assembling the full cell, the cathode and the anode was activated in the corresponding half cells. The voltage window for the full battery is 1 to 4.2 V. In addition, the NVP electrodes were fabricated from a mixture of active material, conductive carbon, and PVDF binder, with a mass ratio of 8:1:1. Electrochemical measurements were employed on a LAND 2001A apparatus (Wuhan, China) within the voltage range of 2.0 to 4.0 V (vs. Na/Na^+).

Theoretical Calculation

All DFT calculations were performed using the CP2K 2025.01 package,[3] with computations carried out under periodic boundary conditions. Structural optimization was

performed using the PBE functional with the POB-DZVP-rev2 basis set, and D3 dispersion correction was applied to accurately describe the interactions between COF and ions. Electronic structure calculations were carried out using the HSE06 functional and 6-31G* basis set with only the Γ point considered, yielding the energy level distribution and electrostatic potential. The electrostatic potential was visualized using the VMD program,[4] and charge decomposition analysis (CDA) was performed using the Multiwfn program (version 3.8).[5, 6] The charge density difference between COF and the ions was calculated, and the ELF (Electron Localization Function) color maps were generated with the assistance of this program. The charge density difference maps and molecular orbital diagrams were visualized using the VESTA program.[7] Energy calculations, including frequency calculations, were performed using the HSE06 functional with the 6-311G* basis set, and thermodynamic quantities were derived with the help of the Shermo program.[8]

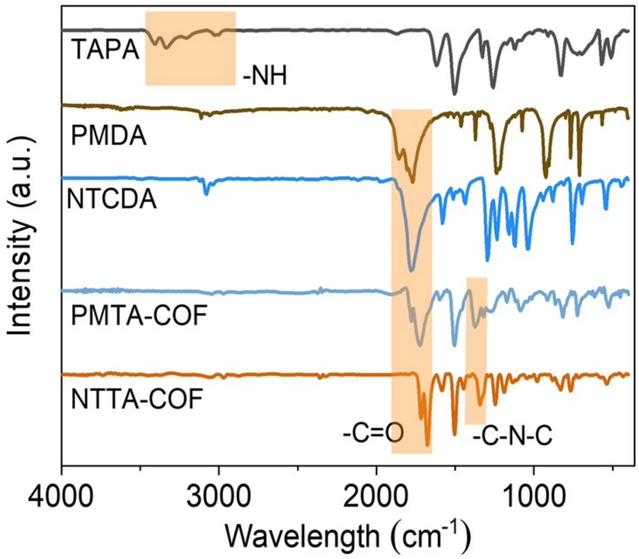

Figure S3. FTIR spectra of TAPA, PMDA, NTCDA, PMTA-COF and NTTA-COF.

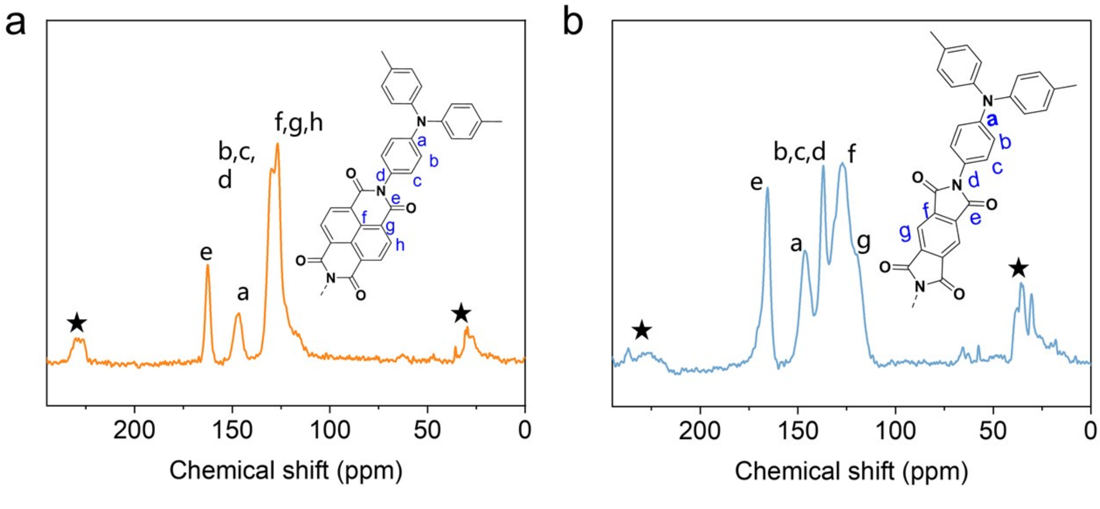

Figure S4. The solid-state ^{13}C CP-MAS NMR spectrum of (a) NTTA-COF and (b) PMTA-COF. The side bands were marked with asterisks.

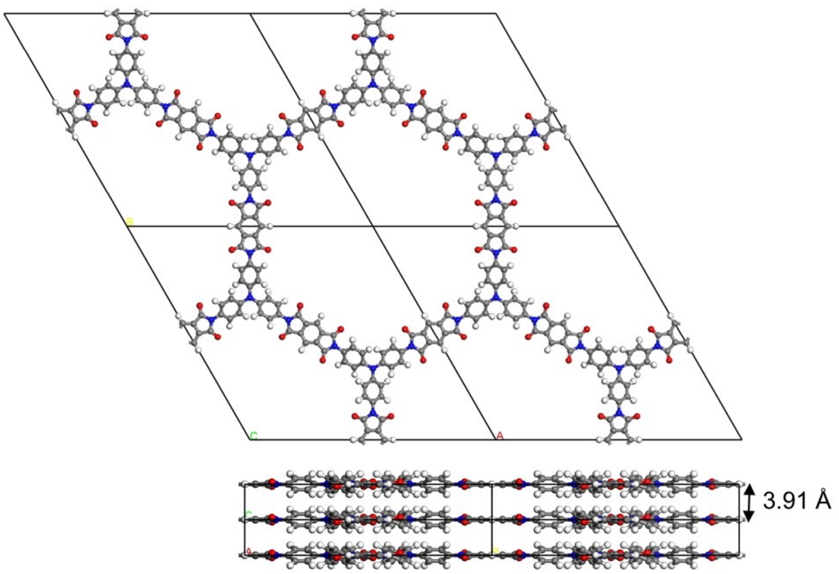

Figure S5. The top and side view of eclipsed AA stacking for PMTA-COF (a= 31.3690 Å, b= 31.3690 Å, c= 3.9100 Å, α=90 º, β= 90 º, γ= 120 º).

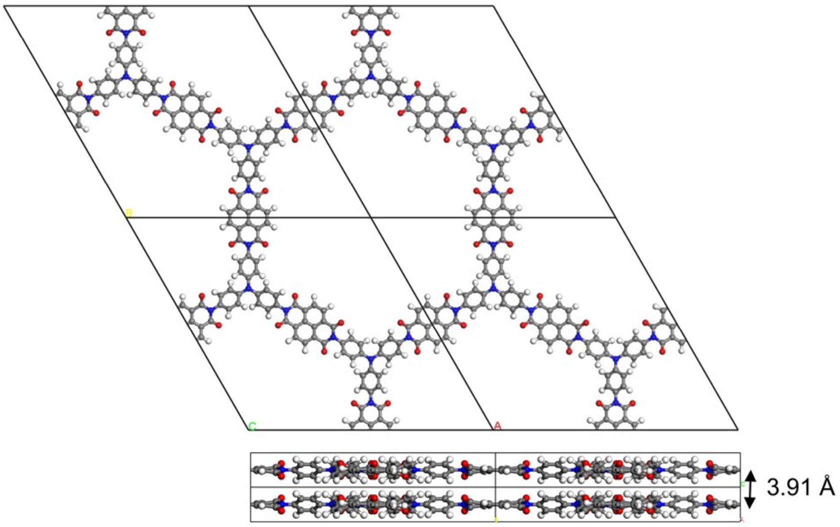

Figure S6. The top and side view of eclipsed AA stacking for NTTA-COF (a= 31.9450 Å, b= 31.9450 Å, c= 3.9010 Å, α=90 º, β= 90 º, γ= 120 º).

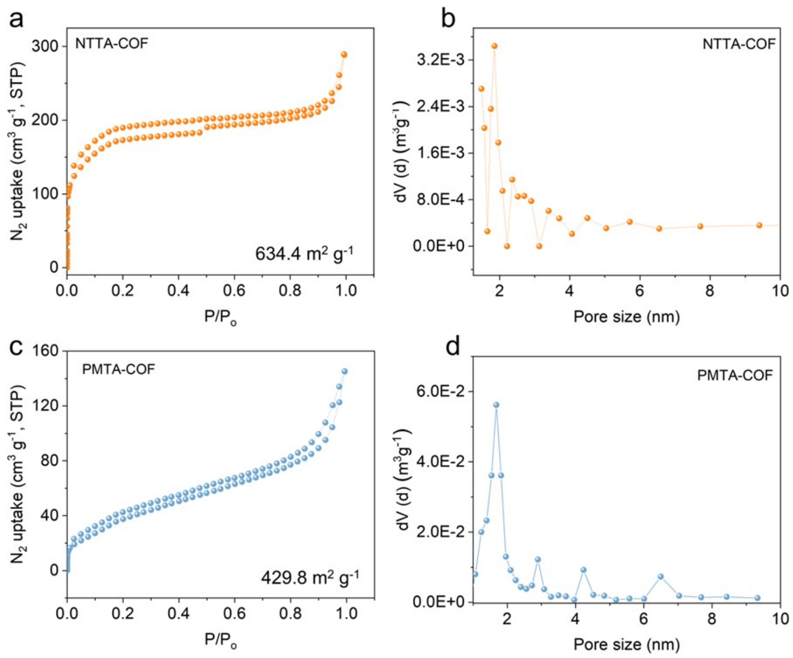

Figure S7. N_2 adsorption-desorption isotherms of (a) NTTA-COF and (c) PMTA-COF. Pore size distribution of (b) NTTA-COF and (d) PMTA-COF.

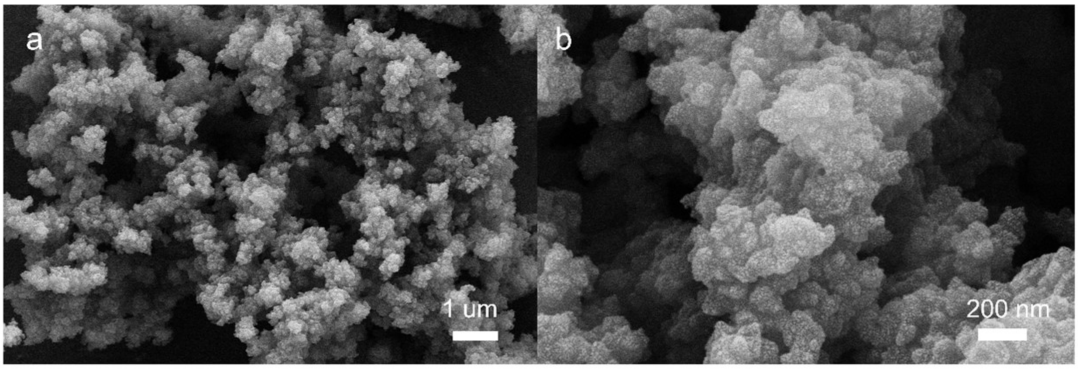

Figure S8. SEM images of NTTA-COF.

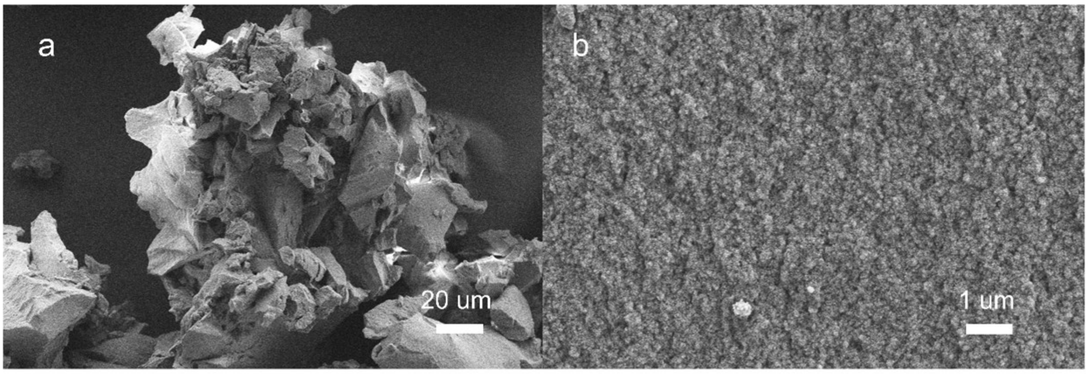

Figure S9. SEM images of PMTA-COF.

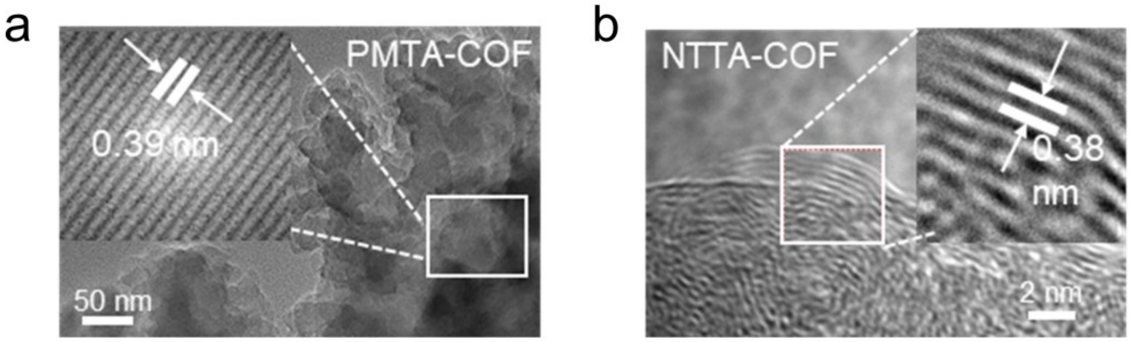

Figure S10. TEM images of (a) NTTA-COF and (b) PMTA-COF.

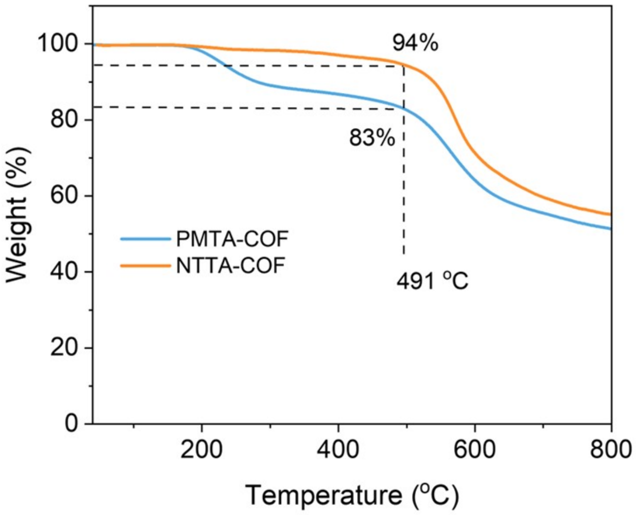

Figure S11. Thermogravimetric analysis (TGA) curves of PMTA-COF and NTTA-COF under nitrogen atmosphere.

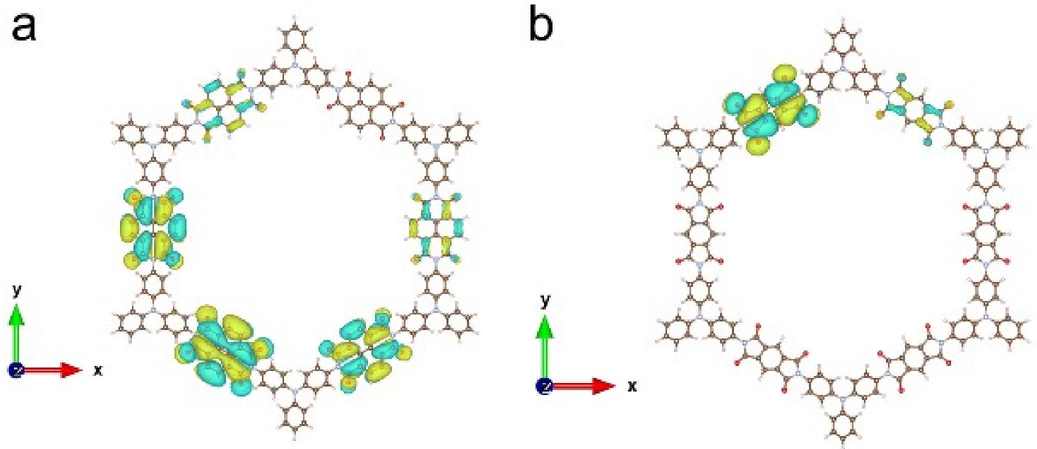

Figure S12. LUMO energy levels of (a) NTTA-COF and (b) PMTA-COF.

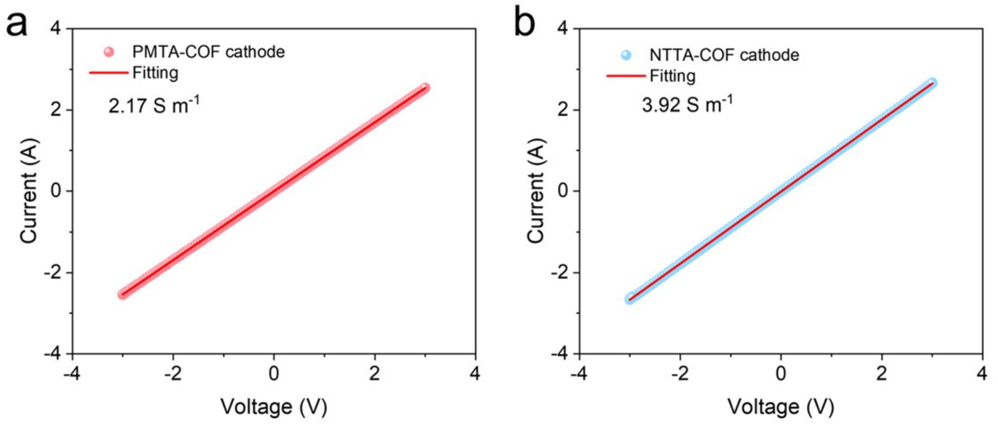

Figure S13. The I-V curves by NTTA-COF and PMTA-COF cathodes of the dual probe.

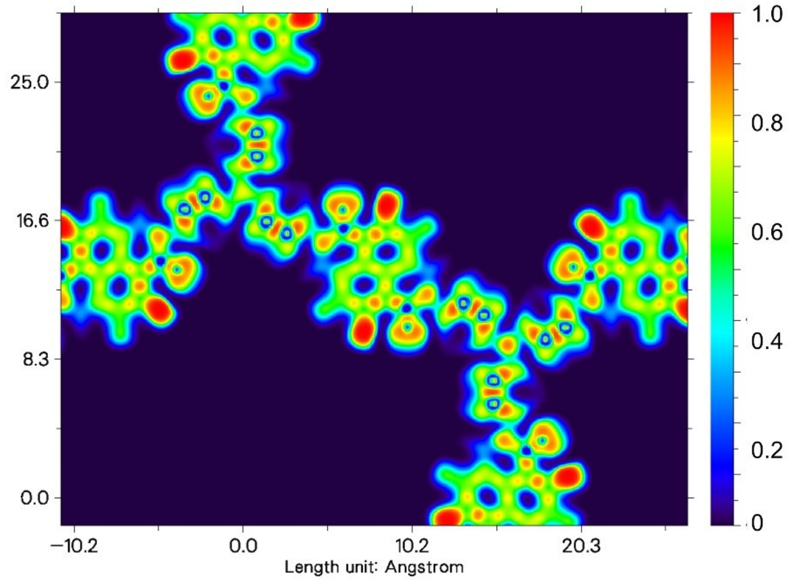

Figure S14. Electron Localization Function for NTTA-COF.

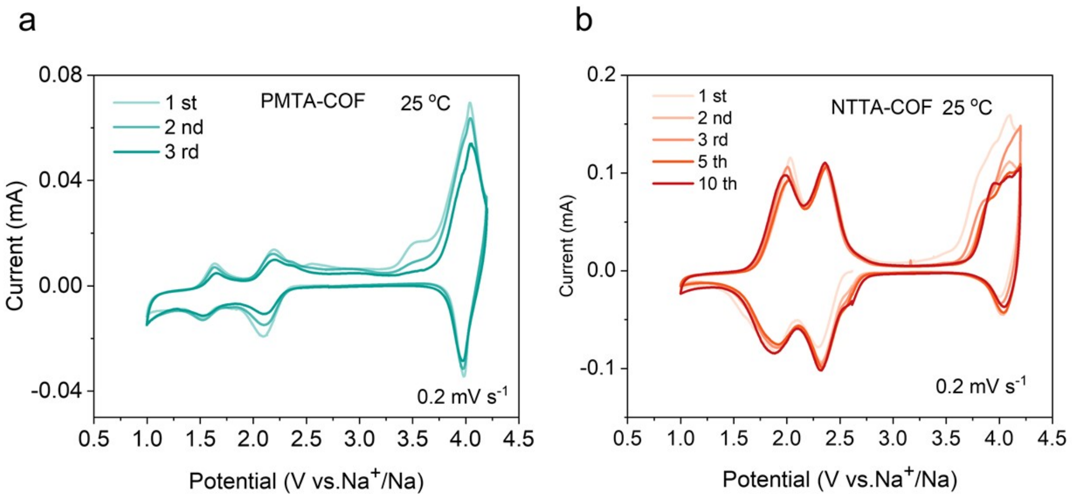

Figure S15. CV curves of (a) PMTA-COF and (b) NTTA-COF at a scan rate of 0.2 mV s^{-1}.

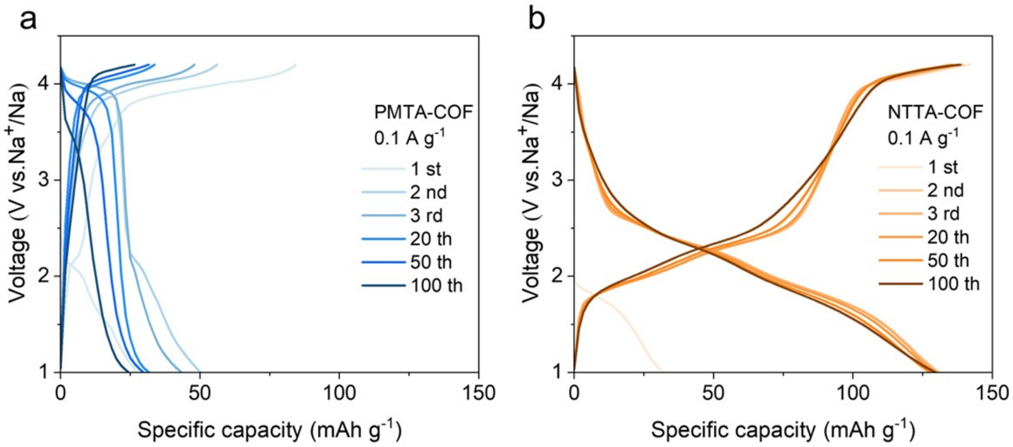

Figure S16. Charge-discharge curves of (a) PMTA-COF and (b) NTTA-COF at a current density of 0.1 A g^{-1}.

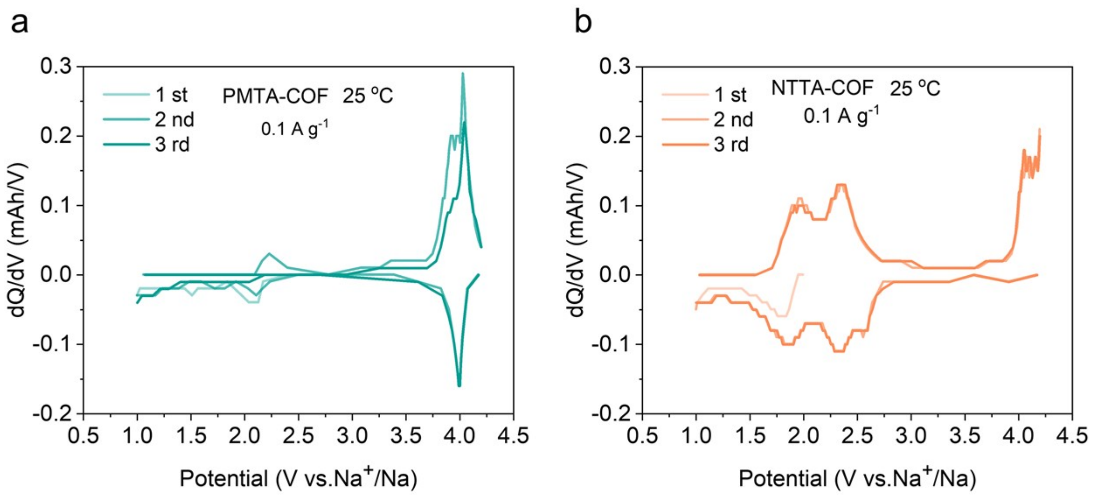

Figure S17. Galvanostatic dQ/dV curve at 0.1 A g^{-1} for (a) PMTA-COF and (b) NTTA-COF.

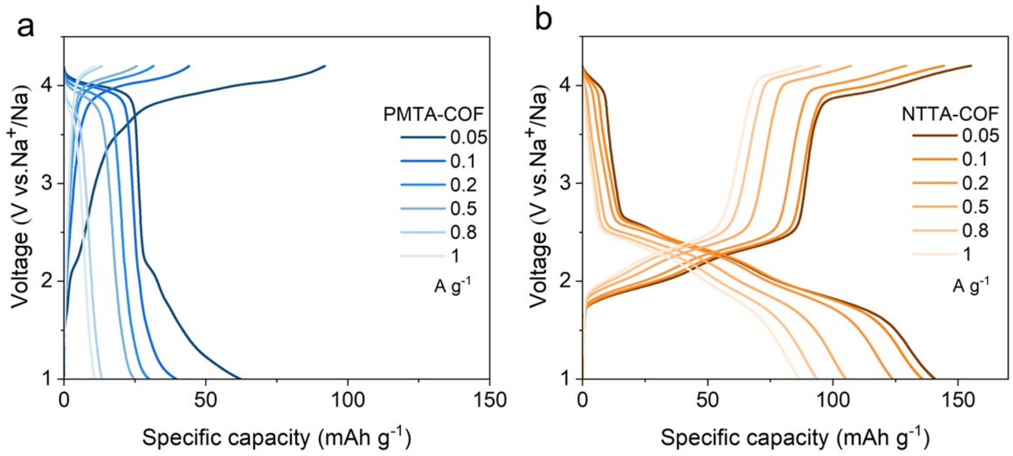

Figure S18. Charge-discharge curves of (a) PMTA-COF and (b) NTTA-COF at various current densities.

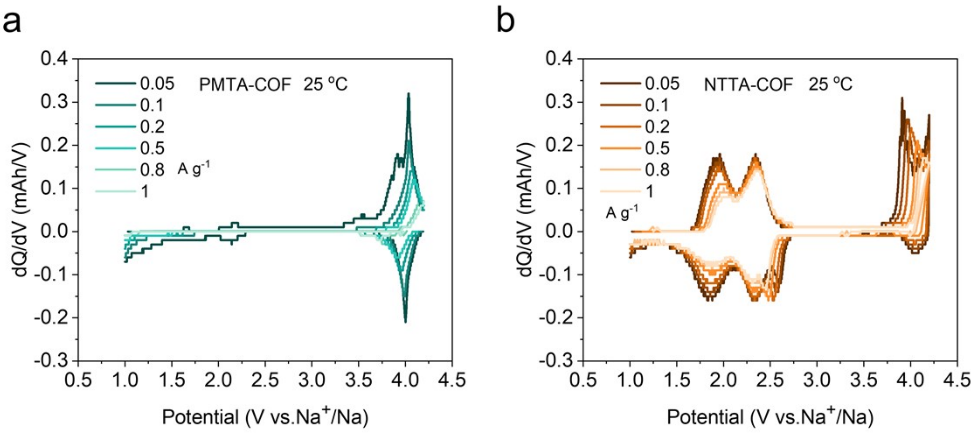

Figure S19. Galvanostatic dQ/dV curves at various current densities for (a) PMTA-COF and (b) NTTA-COF.

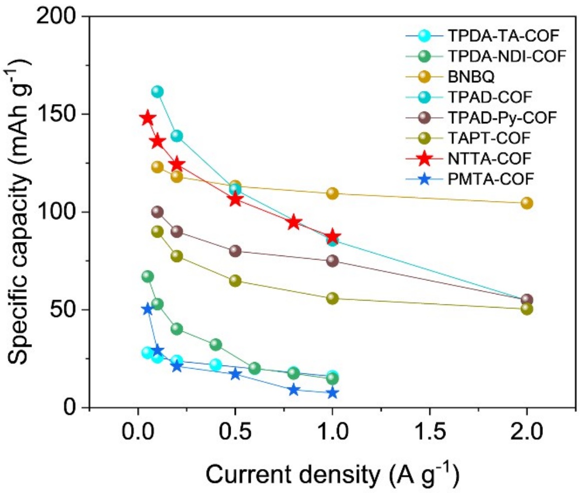

Figure S20. Comparison of the rate performance of NTTA-COF and some reported similar ambipolar COF or polymer materials.

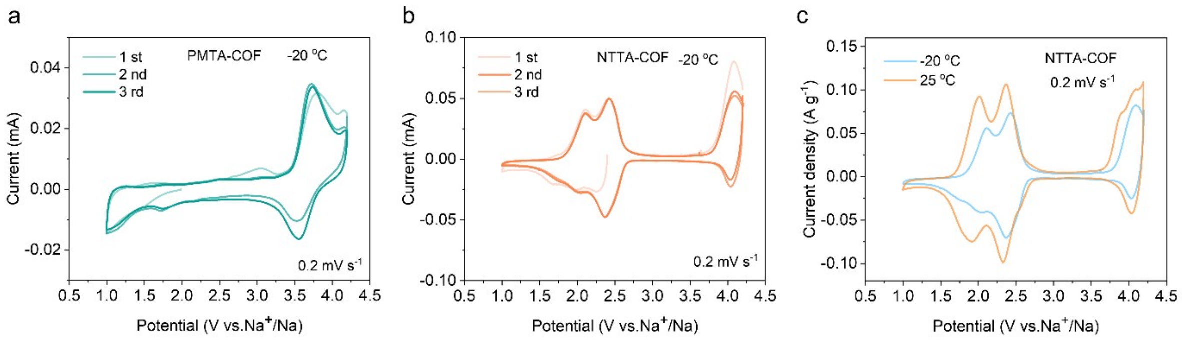

Figure S21.CV curves of (a) PMTA-COF and (b) NTTA-COF at a scan rate of 0.2 mV s^{-1} (-20 °C). (c) CV curves of NTTA-COF at a scan rate of 0.2 mV s^{-1} (-20 °C and 25 °C).

Notably, the oxidation peak at 3.8 V and the reduction peak at 2.6 V for NTTA-COF become indistinct at low temperature. This phenomenon is likely attributed to the sluggish ion diffusion and increased charge-transfer resistance at low temperatures, which intensify the polarization of these redox couples, leading them to be enveloped or masked by the primary redox peaks.

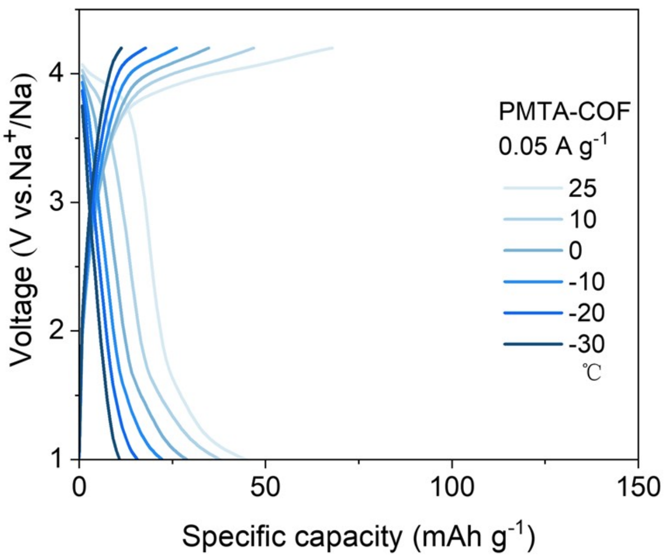

Figure S22. Temperature- dependent charge-discharge curves of PMTA-COF at 0.05 A g^{-1}.

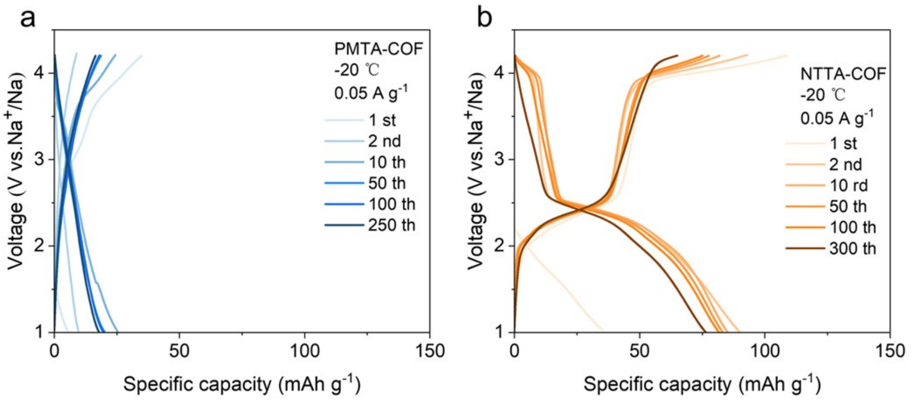

Figure S23. Charge-discharge curves of (a) PMTA-COF and (b) NTTA-COF at 0.05 A g^{-1} (-20 ^{o}C).

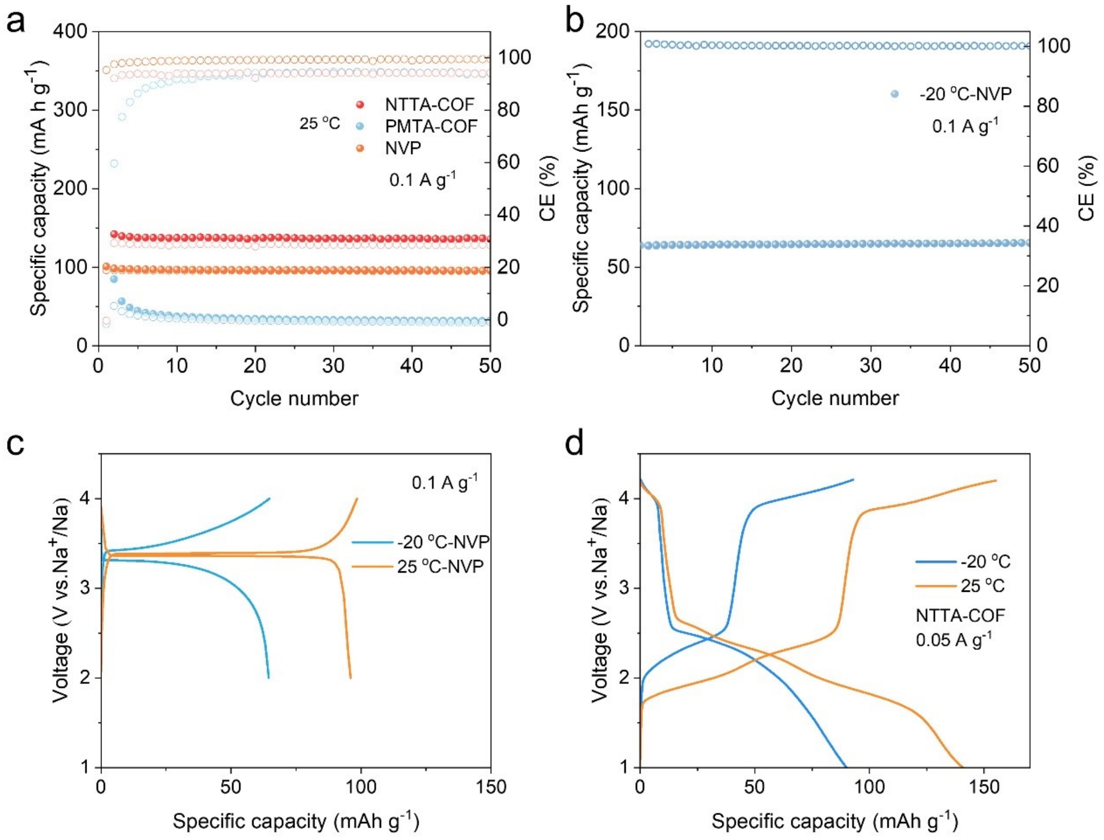

Figure S24. (a) Cycling stability at 0.1 A g^{-1} for NVP, PMTA-COF and NTTA-COF (25 ^{o}C). (b) Cycling stability at 0.1 A g^{-1} for NVP (-20 ^{o}C). (c) Charge-discharge curves of NVP at 0.1A g^{-1} (25 ^{o}C and -20 ^{o}C). (d) Charge-discharge curves of NTTA-COF at 0.05A g^{-1} (25 ^{o}C and -20 ^{o}C).

The electrochemical performance of NVP was further evaluated, as illustrated in Fig. S24. At room temperature with a current density of 0.1 A g^{-1}, the NVP electrode delivers a specific capacity of only 100 mAh g^{-1}. When the temperature decreases to -20 °C, its capacity further declines to 60 mAh g^{-1}, accompanied by an increase in voltage polarization.

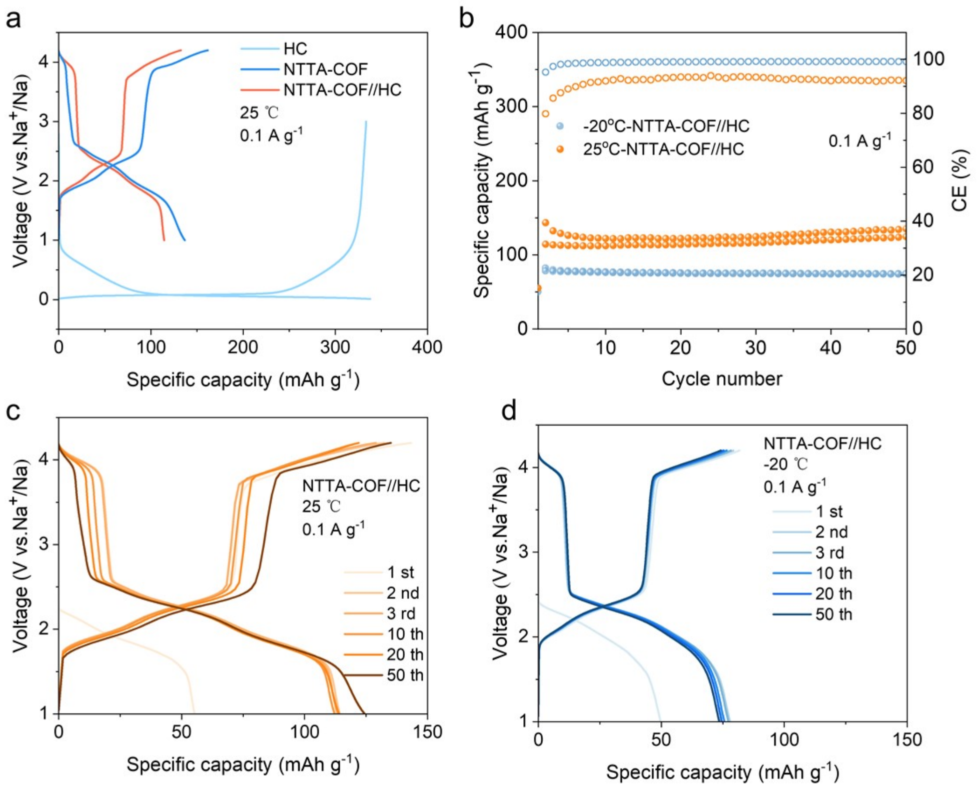

Figure S25. (a) Charge-discharge curves of HC, NTTA-COF and NTTA-COF//HC full cell at 0.1 A g^{-1} (25 ^{o}C). (b) Cycling stability at 0.1 A g^{-1} for NTTA-COF//HC full cell (25 ^{o}C and -20 ^{o}C). Charge-discharge curves of NTTA-COF//HC full cell at (c) 25 ^{o}C and (d) -20 ^{o}C, respectively.

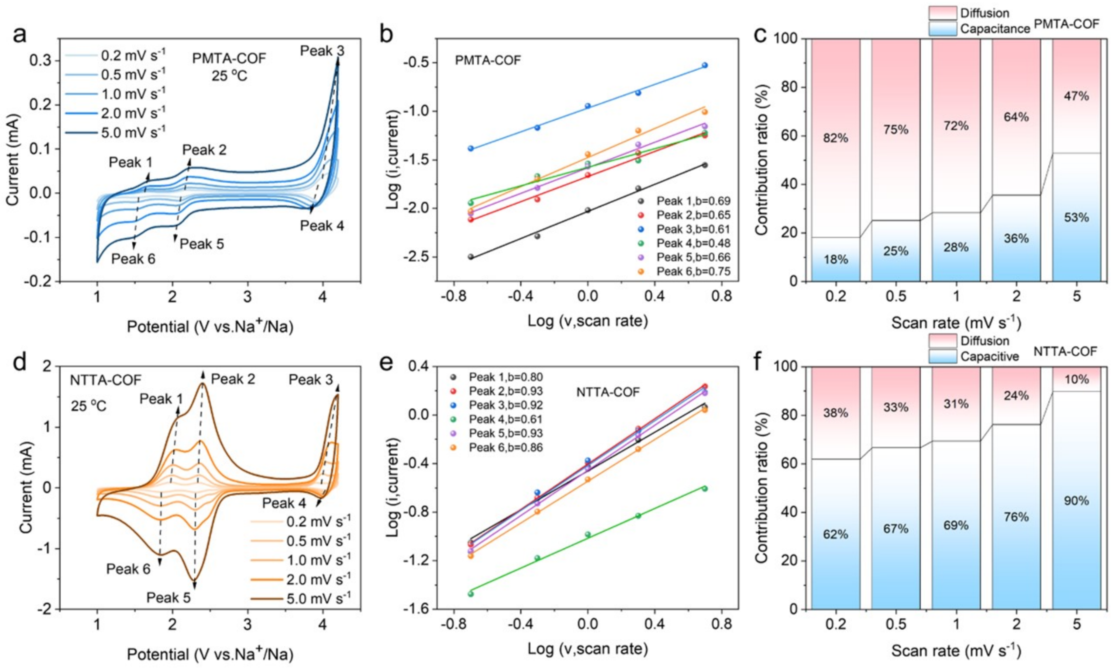

Figure S26. CV curves of (a) PMTA-COF and (c) NTTA-COF at different scan rates (0.2-5.0 mV s^{-1}). The b-values (b) PMTA-COF and (d) NTTA-COF determined from catholic current peaks. The contribution ratio of the pseudocapacitance at various scan rates for (c) PMTA-COF and NTTA-COF.

It is well established that surface-controlled reactions facilitate superior rate capability. The contributions of surface capacitive control and diffusion control to the total capacity at various scan rates were quantitatively investigated using the following equation: $i=k_1v+k_2v^{1/2}$, where $k_1\nu$ and $k_2\nu^{1/2}$ correspond to the capacitive and diffusion contributions, respectively. As illustrated in Figure R1, the capacitive contribution to the total capacity of NTTA-COF was approximately 62% at 0.2 mV s^{-1}, increasing to 90% at 5 mV s^{-1}. In contrast, PMTA-COF exhibited a significantly lower capacitive contribution ratio under identical conditions. This dominance of capacitive behavior endows NTTA-COF with exceptional electrochemical kinetics.

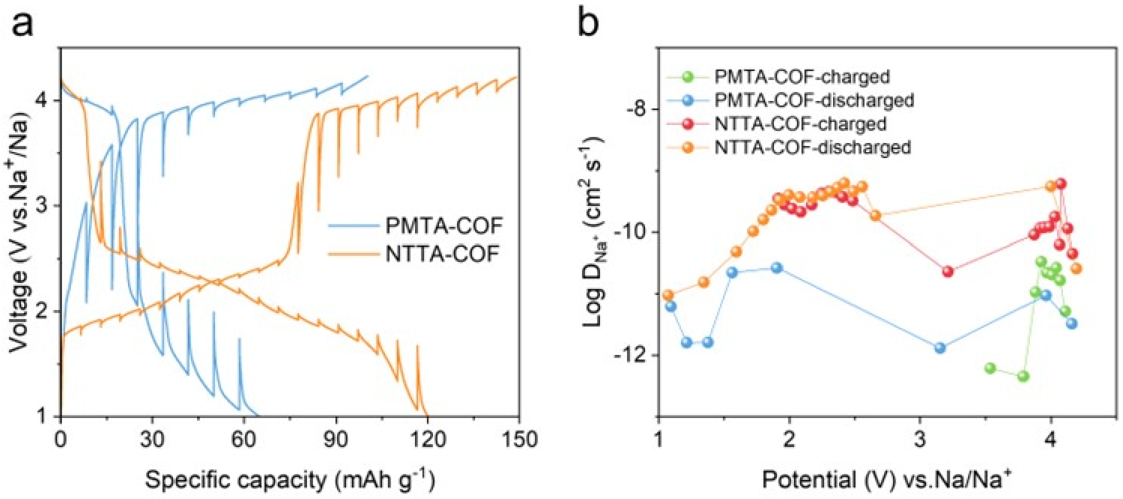

Figure S27. (a) GITT curves of PMTA-COF and NTTA-COF electrodes, respectively. (b) Na-ion diffusion coefficient for PMTA-COF and NTTA-COF electrodes, respectively.

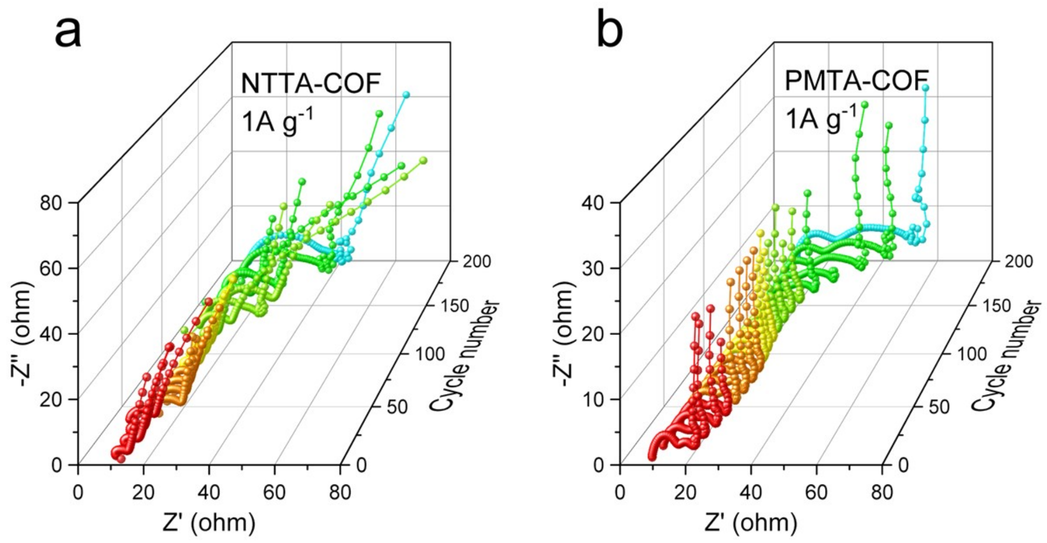

Figure S28. In-situ Nyquist plots collected at various cycle numbers for (a) NTTA-COF and (b) PMTA-COF (at 1 A g^{-1}).

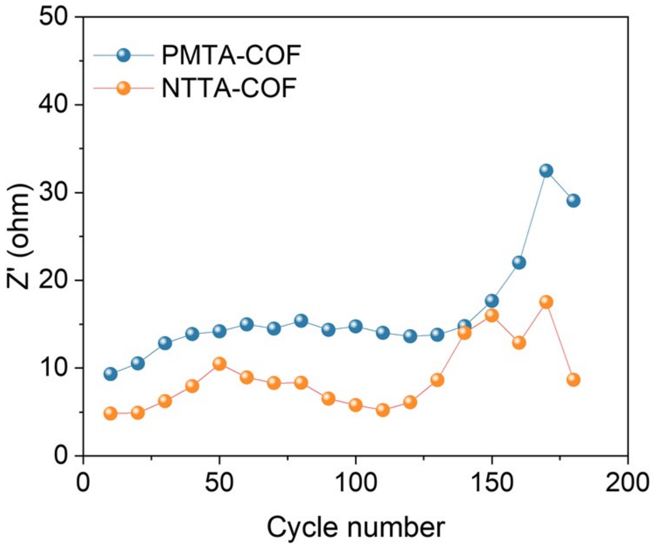

Figure S29. The impedance values collected from PMTA-COF and NTTA-COF electrodes at different cycles.

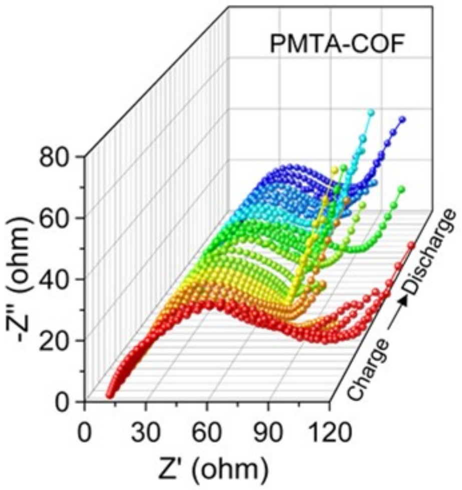

Figure S30. In-situ Nyquist plots collected at various charged and discharged states of PMTA-COF.

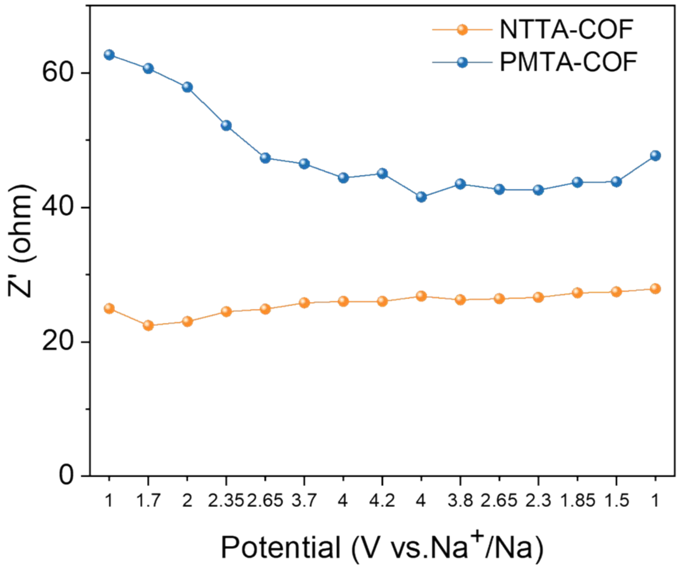

Figure 31. Comparison of EIS values of NTTA-COF and PMTA-COF during the charge-discharge process.

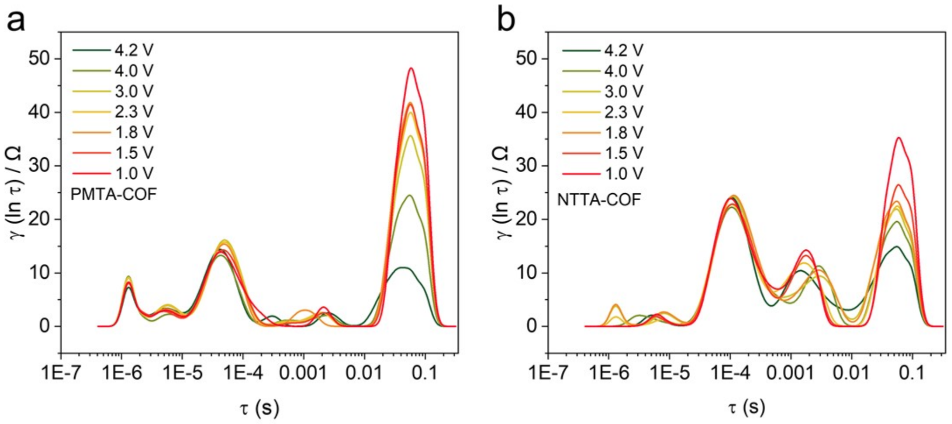

Figure S32. In situ DRT profiles for (a) PMTA-COF and (b) NTTA-COF.

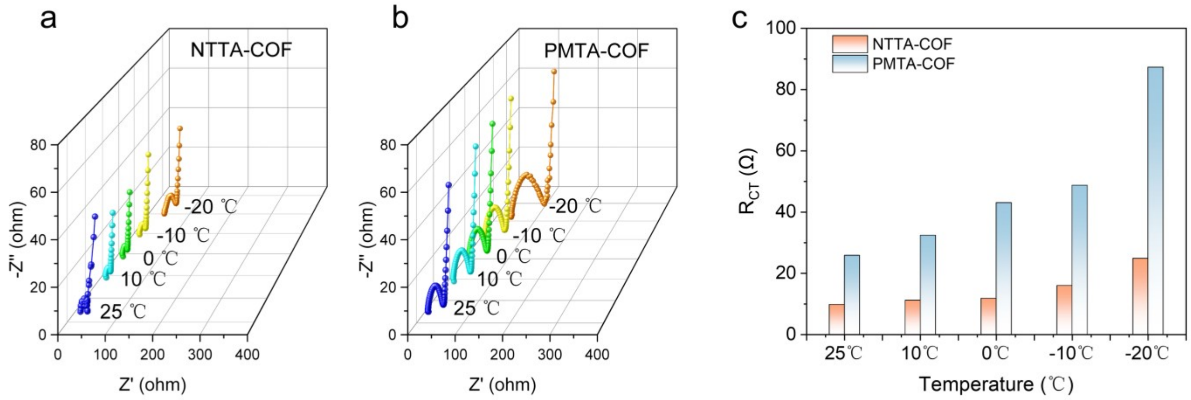

Figure S33. In-situ Nyquist plots collected at various temperatures for (a) NTTA-COF and (b) PMTA-COF. (c) The R_{CT} at various temperatures for PMTA-COF and NTTA-COF.

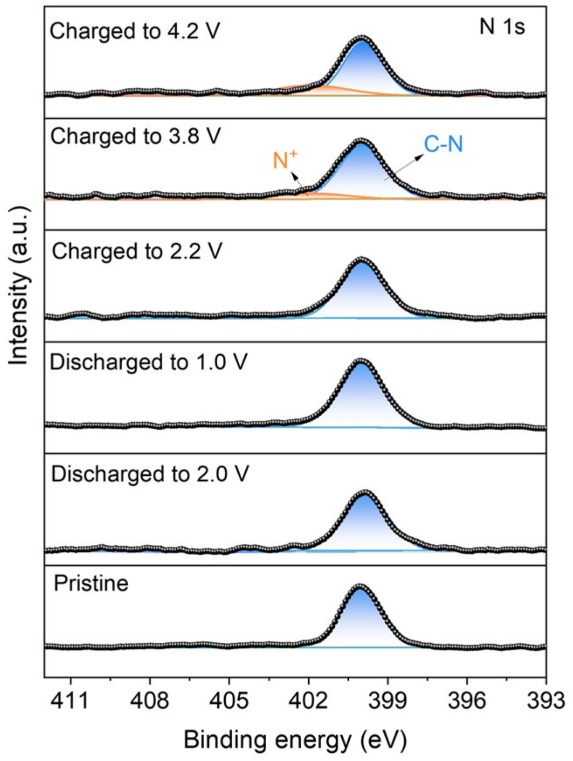

Figure S34. High-resolution N 1s XPS spectra of NTTA-COF.

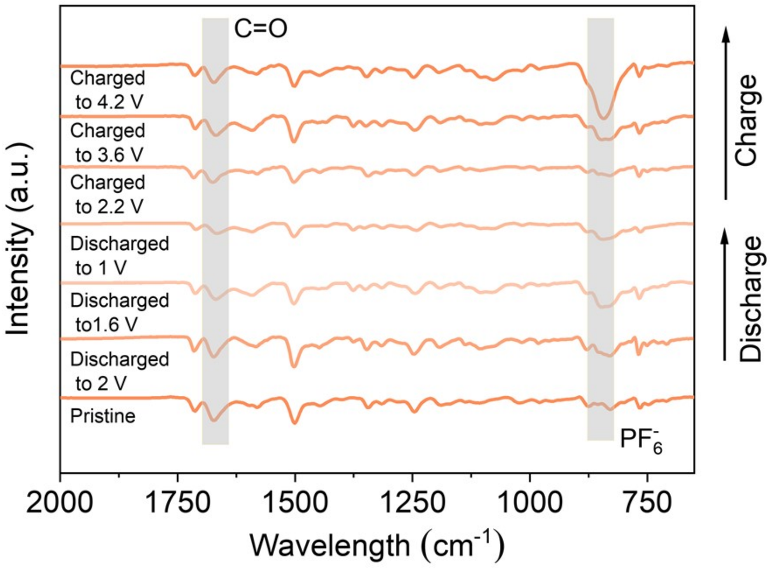

Figure S35. Ex situ FTIR spectra for NTTA-COF.

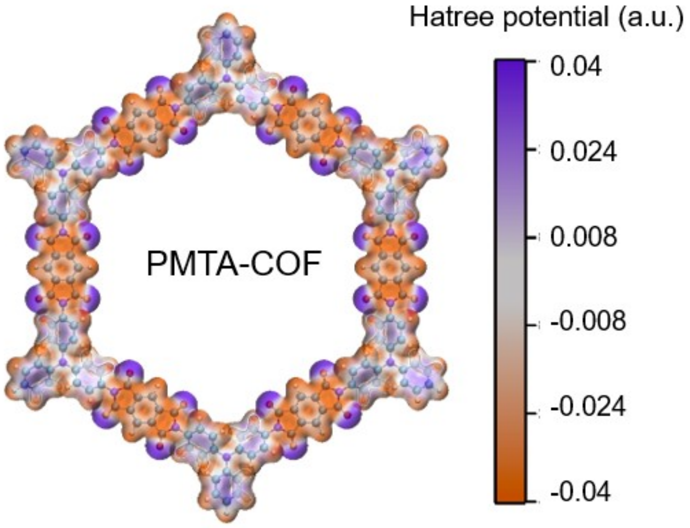

Figure S36. Electrostatic potential diagram of PMTA-COF.

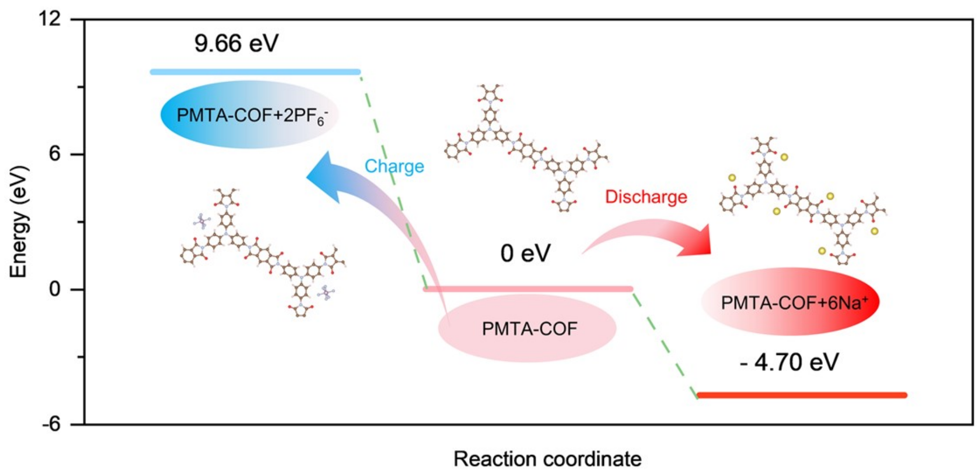

Figure S37. Calculated energies of Na-ion during the possible redox routes of PMTA-COF.

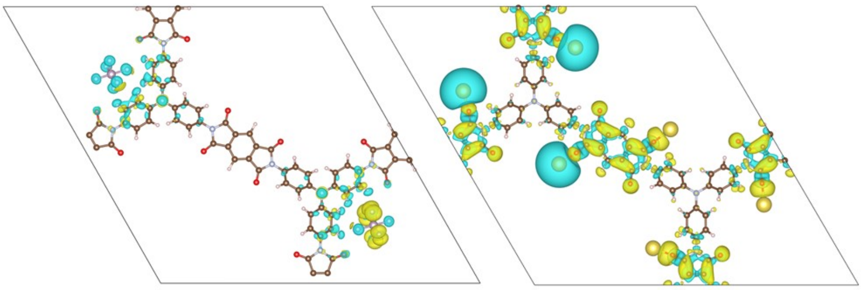

Figure S38. Charge density difference of PMTA-COF+2PF_6^- and PMTA-COF+6Na^+.

Table S1. Crystallography parameters of MTA-COF and NTTA-COF.

Crystallography parameters	PMTA-COF	NTTA-COF
a (Å)	31.3690	31.9450
b (Å)	31.3690	31.9450
c (Å)	3.9100	3.9010
α	90 °	90 °
β	90 °	90 °
γ	120 °	120 °

Table S2. Comparison of the thermal stability of NTTA-COF and some reported similar ambipolar COF or polymer materials.

Materials	Onset decomposition temperature (T_{onset}/°C)	Weight retention at T_{onset} (%)	Ref.
NTTA-COF 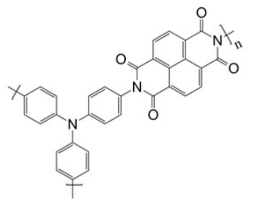	491	94	This work
PMTA-COF 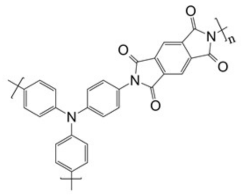	491	83	This work
APCNDI 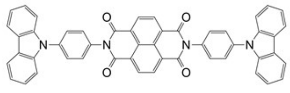	~450	~90	[9]
TPDA-TA-COF 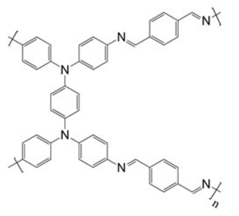	~420	~95	[10]
TPDA-NDI-COF 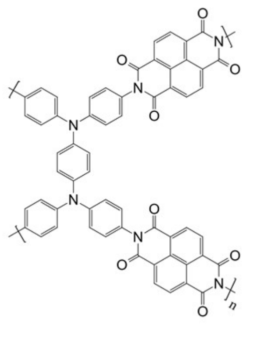	~450	~90	[10]

TPDA-NDI-COF-50%CNT	~450	~92	10
TAC 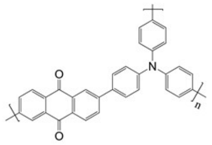	~600	~95	11
BNBQ 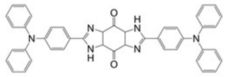	412	90	12
TPAD-COF 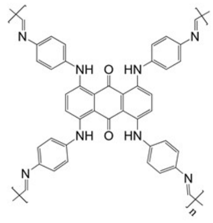	400	~95	13
TPAD-Py-COF 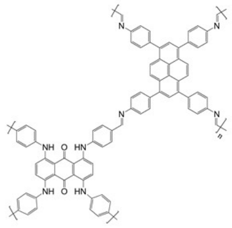	508	~95	13
BTT-PTO-COF 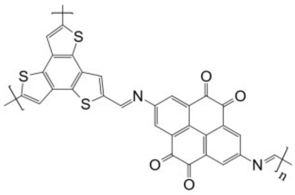	350	~90	14
TPDA-PMDA-COF 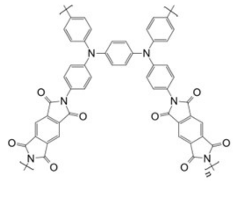	~600	~95	15

PyNTB-COF 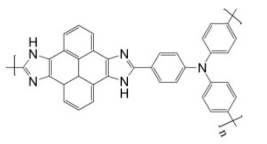	300	~95	16
HATN-AQ-COF 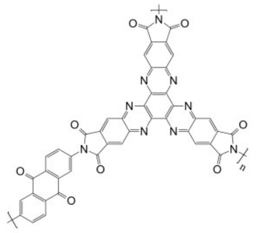	451	~98	17

Table S3. Electrochemical performance of reported COFs as cathode for SIBs.

Electrodes	Operation voltage window (V)	Average discharge Voltage (V)	Reversible Capacity/ mAh g^{-1} (current, mA g^{-1})	Capacity retention (cycle number, current density (mA g^{-1}))	Ref.
NTTA-COF	1-4.2	2.3	131 (100)	80% (2000, 1000)	This work
PMTA-COF	1-4.2	2.1	50.3 (100)	48% (100, 100)	This work
TQBQ-COF	1-3.6	2	327.2 (100)	91.3% (1000, 500)	18
HATN-HHTP@CNT	1-3.4	1.75	225 (50)	82% (5000, 1000)	19
HATN-HHTP	1-3.4	1.75	~120 (50)	-	
TP-Pa-COF	0.8-3.8	1.2	~75 (100)	~25% (200, 100)	20
TP-OH-COF	0.8-3.8	2.2	96.04 (100)	100% (200, 100)	
TP-OH-COF@CNT50	0.8-3.8	2.2	207.6 (100)	100% (200, 100)	
BAV-COF:Cl^-	1.4-3.9	2.2	150 (50)	20% (500, 250)	21
BAV-COF:Br^-	1.4-3.9	3.3<br>2.2	165 (50)	84.2% (500, 250)	
BAV-COF:I^-	1.4-3.9	3.2<br>2.2	160 (50)	70.6% (500, 250)	
BAV-COF:ClO_4^-	1.4-3.9	2.2	138 (50)	19% (500, 250)	
BPOE	1.3-4.1	~2	~200 (100)	80% (7000, 1000)	22
TAPA-COF	1.5-3.2	2.1	~90 (100)	~80% (100, 500)	23
S@TAPA-COF	1.5-3.2	2.1	~125 (100)	68% (100, 500)	
TAPT-COF	1.5-3.2	2	69.4 (500)	83% (100, 500)	
S@TAPT-COF	1.5-3.2	2	109.3 (100)	75% (100, 500)	
HATN-PD-COF	1-3.6	1.9	210 (200)	91% (7000, 10000)	24
HATN-TAB-COF	1-3.6	1.8	150 (200)	93% (3000, 10000)	

TPDA-NDI-COF	1-3.6	3.2<br>2	78<br>(20)	89.7%<br>(300, 20)	10
TPDA-NDI-COF-50%CNT	1-3.6	3.2<br>2	120<br>(20)	97.8%<br>(300, 20)	
TPDA-TA-COF	1-3.6	3.2<br>2	28<br>(50)	-	
TAPB-NDI	1.5-3.5	2.2	25<br>(25)	~80%<br>(50, 25)	25
TAPB-NDI@CNT50	1.5-3.5	2.2	136.7<br>(25)	80%<br>(100, 300)	
TP-PDA	1.3-4.2	3.1	175<br>(100)	100%<br>(1000, 1000)	26

Table S4. Rate performance of NTTA-COF and some reported similar ambipolar COF or polymer materials.

**Electrodes**	**Specific capacity,current (mAh g^{-1}, A g^{-1})**	**Rate performanceCurrent density (A g^{-1}) Capacity retention (%)**	**Ref.**
NTTA-COF	148, 0.1	0.05,0.1,0.2,0.5,0.8,1<br>148,136,124,106,94,87	This work
PMTA-COF	50.3, 0.1	0.05,0.1,0.2,0.5,0.8,1<br>50.3,29,21,17,9,7.5	This work
APCNDI	141, 0.1	0.1,0.2,0.5,1,2,5,10<br>141,135,129,120,110,100,70	[9]
TPDA-TA-COF	28,0.05	0.05,0.1,0.2,0.4,0.6,0.8,1<br>28,26,24,22,20,18,16	[10]
TPDA-NDI-COF	67,0.05	0.05,0.1,0.2,0.4,0.6,0.8,1<br>67,53,40,32,20,17,15	[10]

TPDA-NDI-COF-50%CNT	120,0.05	0.05,0.1,0.2,0.4,0.6,0.8,1 120,114,104,97,95,90,87	10
TP-PDA 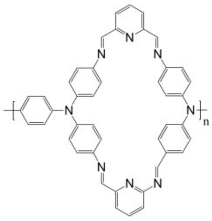	175,0.1	0.1,0.3,0.5,1,3,5 175,140,124,108,100,89	26
TAC	193, 0.5	0.5,1,2,4,10,15,20 193,177,164,152,131,121,102	11
BNBQ	123.1, 0.1	0.1,0.2,0.5,1,2 123,117,113,109,104	12
TPAD-COF	161.5, 0.1	0.1,0.2,0.5,1,2 161,138,111,85,54	13
TPAD-Py-COF	100, 0.1	0.1,0.2,0.5,1,2 100,90,80,75,55	13

TAPB-NDA@CNT50 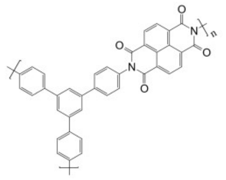	124, 0.025	0.025,0.05,0.1,0.5,1,2,5<br>124,109,97,84,66,62,48	25
TAPA-COF 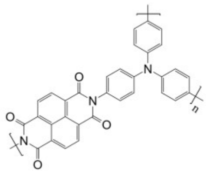	75, 0.1	0.1,0.2,0.5,1,2<br>75,70,60,55,50	23
TAPT-COF 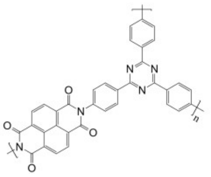	90, 0.1	0.1,0.2,0.5,1,2<br>90,77,65,56,50	23
S@TAPA-COF 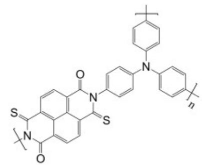	105, 0.1	0.1,0.2,0.5,1,2<br>105,89,78,65,60	23
S@TAPT-COF 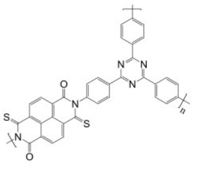	109.3, 0.1	0.1,0.2,0.5,1,2<br>109,88,78,73,67	23

Table S5. Electrochemical performance of reported COFsfor SIBs in low temperature.

Electrode	Operation voltage window (V)	Mean discharge voltage (V)	Temperature (°C)	Reversible Capacity at low temperature/ mAh g^{-1} (current, mA g^{-1})	Capacity retention at low temperature (cycle number, current density (mA g^{-1}))	Ref.
NTTA-COF	1-4.2	2.3	-20	85 (100)	91% (300, 50)	This work
PMTA-COF	1-4.2	2.1	-20	50.3 (100)	74% (275, 50)	This work
TRO-PMDA-COF	0.01-2.8	~0.6	-30	75 (50)	-	27
TRO-PMDA-COF@CNT	0.01-2.8	~0.6	-30	156.8 (50)	97% (100, 50)	
TPAD-COF	1.5-4.2	~2.5	-20	75 (100)	~100 % (300, 100)	13
nDSR*π	1-3.5	~2.1	-50	130 (50)	~100 % (65, 50)	28
HATN-O-Zn	0.01-3.0	~0.3	-20	98 (100)	84.4% (200, 100)	29
PTCDA/PPy/CNTs	1.9-3.0	2.28	-30	86.5 (1C)	79.4% (500, 5C)	30
DABT	1.2-2.8	~1.5	-20	125.5 (0.2C)	80% (100, 0.2C)	31
$NTONb_{0.08}$	1.01-2.5	~0.5	-15	103 (100)	~100 % (200, 100)	32
$Na_3V_2(PO_4)_2O_2F$	2.5-4.2	~3.5	-40	71 (65)	24% (100, 130)	33
CB-$Na_3V_2(PO_4)_2O_2F$	2.5-4.2	~3.5	-40	88 (65)	88.4% (100, 130)	
$Na_3V_2(PO_4)_3$	2.3-4.0	~3.0	-40	80 (0.1C)	88.2% (100, 0.1C)	34
$NaCrO_2$	2.0-3.6	~3.0	-10	90 (24)	97.2% (100, 120)	35
$Na_3V_{1.98}Mn_{0.02}(PO_4)_2F_3$	2.0-4.3	~3.7	-25	80 (0.5C)	93.3% (400, 0.5C)	36
$Na_{0.78}Ni_{0.31}Mn_{0.67}Nb_{0.02}O_2$	2.4-4.2	~3.2	-40	88 (92)	76% (1800, 368)	37
$Na_2Ti_3O_7$	0-2.5	~0.4	-40	50 (100)	~100 % (3000, 100)	38

Table S6. The EIS values of NTTA-COF and PMTA-COF during the charge-discharge process.

**Voltage (V vs. Na/Na$^+$)**	**NTTA-COF Z' (ohm)**	**PMTA-COF Z' (ohm)**
Discharged to 1.0	24.95	62.68
Charged to 1.7	22.43	60.64
Charged to 2.0	23.02	57.88
Charged to 2.35	24.48	52.17
Charged to 2.65	24.87	47.34
Charged to 3.7	25.8	46.49
Charged to 4.0	26.01	44.39
Charged to 4.2	26.03	45.03
Discharged to 4.0	26.79	41.55
Discharged to 3.8	26.26	43.47
Discharged to 2.65	26.42	42.67
Discharged to 2.3	26.61	42.57
Discharged to 1.85	27.28	43.72
Discharged to 1.5	27.44	43.81
Discharged to 1.0	27.9	47.69

References

1 P.-H. Chang, M. C. Sil, K. S. K. Reddy, C.-H. Lin and C.-M. Chen, Polyimide-Based Covalent Organic Framework as a Photocurrent Enhancer for Efficient Dye-Sensitized Solar Cells, ACS Appl. Mater. Interfaces., 2022, 14, 25466-25477.

2 G. Wang, N. Chandrasekhar, B. P. Biswal, D. Becker, S. Paasch, E. Brunner, M. Addicoat, M. Yu, R. Berger and X. Feng, A Crystalline, 2D Polyarylimide Cathode for Ultrastable and Ultrafast Li Storage, Adv. Mater., 2019, 31, 1901478.

3 T. D. Kühne, M. Iannuzzi, M. Del Ben, V. V. Rybkin, P. Seewald, F. Stein, T. Laino, R. Z. Khaliullin, O. Schütt, F. Schiffmann, D. Golze, J. Wilhelm, S. Chulkov, M. H. Bani-Hashemian, V. Weber, U. Borštnik, M. Taillefumier, A. S. Jakobovits, A. Lazzaro, H. Pabst, T. Müller, R. Schade, M. Guidon, S. Andermatt, N. Holmberg, G. K. Schenter, A. Hehn, A. Bussy, F. Belleflamme, G. Tabacchi, A. Glöß, M. Lass, I. Bethune, C. J. Mundy, C. Plessl, M. Watkins, J. VandeVondele, M. Krack and J. Hutter, CP2K: An electronic structure and molecular dynamics software package - Quickstep: Efficient and accurate electronic structure calculations, J. Chem. Phys., 2020, 152, 194103.

4 W. Humphrey, A. Dalke and K. Schulten, VMD: Visual molecular dynamics, J. Mol. Graph., 1996, 14, 33-38.

5 T. Lu and F. Chen, Multiwfn: A multifunctional wavefunction analyzer, J. Comput. Chem., 2011, 33, 580-592.

6 T. Lu, A comprehensive electron wavefunction analysis toolbox for chemists, Multiwfn, J. Chem. Phys., 2024, 161, 082503.

7 K. Momma and F. Izumi, VESTA: a three-dimensional visualization system for electronic and structural analysis, J. Appl. Crystallogr., 2008, 41, 653-658.

8 T. Lu and Q. Chen, Shermo: A general code for calculating molecular thermochemistry properties, Comput. Theor. Chem., 2021, 1200, 113249.

9 W. Wang, C. Zhao, J. Yang, P. Xiong, H. Su and Y. Xu, In-situ electropolymerized bipolar organic cathode for stable and high-rate lithium-ion batteries, Sci China Mater 2021, 64, 2938-2948.

10 S. Jindal, Z. Tian, A. Mallick, S. Kandambeth, C. Liu, P. M. Bhatt, X. Zhang, O. Shekhah, H. N. Alshareef and M. Eddaoudi, p/n-Type Polyimide Covalent Organic Frameworks for High-Performance Cathodes in Sodium-Ion Batteries, Small, 2024, 21, 2407525.

11 H. Wang, G. Liu, W. Zhou, Y. Wang and X. Dong, High-Potential and Stable Organic Cathode for Rechargeable Batteries with Fast-Charging and Wide-Temperature Adaptability, Angew. Chem. Int. Ed., 2024, 64, e202416874.

12 L. Zheng, J. Ren, H. Ma, M. Yang, X. Yan, R. Li, Q. Zhao, J. Zhang, H. Fu, X. Pu, M. Hu and J. Yang, Bis-imidazole ring-containing bipolar organic small molecule cathodes for high-voltage and ultrastable lithium-ion batteries, J. Mater. Chem. A., 2023, 11, 108-117.
13 L. Cheng, X. Yan, J. Yu, X. Zhang, H. G. Wang, F. Cui and Y. Wang, Redox-Bipolar Covalent Organic Framework Cathode for Advanced Sodium-Organic Batteries, Adv. Mater., 2025, 37, 2411625.
14 C. Li, A. Yu, W. Zhao, G. Long, Q. Zhang, S. Mei and C. J. Yao, Extending the π-Conjugation of a Donor-Acceptor Covalent Organic Framework for High-Rate and High-Capacity Lithium-Ion Batteries, Angew. Chem. Int. Ed., 2024, 63, e202409421
15 L. Yao, C. Ma, L. Sun, D. Zhang, Y. Chen, E. Jin, X. Song, Z. Liang and K.-X. Wang, Highly Crystalline Polyimide Covalent Organic Framework as Dual-Active-Center Cathode for High-Performance Lithium-Ion Batteries, J. Am. Chem. Soc., 2022, 144, 23534-23542.
16 T.-X. Luan, L.-B. Xing, N. Lu, X.-L. Li, S. Kong, W. W. Yu, P.-Z. Li and Y. Zhao, Donor–Acceptor-π-Acceptor–Donor-Type Photosensitive Covalent Organic Framework for Effective Photocatalytic Aerobic Oxidation, J. Am. Chem. Soc., 2025, 147, 12704-12714.
17 X. Yang, L. Gong, X. Liu, P. Zhang, B. Li, D. Qi, K. Wang, F. He and J. Jiang, Mesoporous Polyimide-Linked Covalent Organic Framework with Multiple Redox-Active Sites for High-Performance Cathodic Li Storage, Angew. Chem. Int. Ed., 2022, 61, e202207043.
18 R. Shi, L. Liu, Y. Lu, C. Wang, Y. Li, L. Li, Z. Yan and J. Chen, Nitrogen-rich covalent organic frameworks with multiple carbonyls for high-performance sodium batteries, Nat. Commun., 2020, 11, 178.
19 S. Li, Y. Liu, L. Dai, S. Li, B. Wang, J. Xie and P. Li, A stable covalent organic framework cathode enables ultra-long cycle life for alkali and multivalent metal rechargeable batteries, Energy Storage Mater., 2022, 48, 439-446.
20 W. Yuan, J. Weng, M. Ding, H.-M. Jiang, Z. Fan, Z. Zhao, P. Zhang, L.-P. Xu and P. Zhou, Novel covalent organic framework/carbon nanotube composites with multiple redox-active sites for high-performance Na storage, Energy Storage Mater., 2024, 65, 103142.
21 Z. Tong, H. Wang, T. Kang, Y. Wu, Z. Guan, F. Zhang, Y. Tang and C.-S. Lee, Ionic covalent organic frameworks with tailored anionic redox chemistry and selective ion transport for high-performance Na-ion cathodes, J. Energy Chem., 2022, 75, 441-447.
22 K. Sakaushi, E. Hosono, G. Nickerl, T. Gemming, H. Zhou, S. Kaskel and J. Eckert, Aromatic porous-honeycomb electrodes for a sodium-organic energy storage device, Nat. Commun., 2013, 4, 1485.

23 J. Shi, W. Tang, B. Xiong, F. Gao and Q. Lu, Molecular design and post-synthetic vulcanization on two-dimensional covalent organic framework@rGO hybrids towards high-performance sodium-ion battery cathode, Chem. Eng. J., 2023, 453, 139607.

24 X. Yang, L. Gong, Z. Liu, Q. Zhi, B. Yu, X. Chen, K. Wang, X. Li, D. Qi and J. Jiang, Hydrothermal synthesis of polyimide-linked covalent organic frameworks towards ultrafast and stable cathodic sodium storage, Sci. Chi. Chem., 2024, 67, 1300-1310.

25 S. Biswas, A. Pramanik, A. Dey, S. Chattopadhyay, T. S. Pieshkov, S. Bhattacharyya, P. M. Ajayan and T. K. Maji, 2D Covalent Organic Framework Covalently Anchored with Carbon Nanotube as High-Performance Cathodes for Lithium and Sodium-Ion Batteries, Small, 2024, 20, 2406173.

26 S. Liu, P. Leung, Y. Zuo, M. Sun, L. Wei, F. C. Walsh, T. Zhao and Q. Liao, High Voltage Flexible Sodium-Ion Battery Cathode Materials Based on 1D Covalent Organic Framework, Adv. Sci., 2025, 12, e05311.

27 S. Yang, J. Wu, Z. Shan, X. Zhang, J. Chu, Y. Chen and C. Wang, Truxenone-Based Covalent Organic Framework/Carbon Nanotube Composite for High-Performance Low-Temperature Sodium-Ion Batteries, Angew. Chem. Int. Ed., 2025, e202511714.

28 X. Xu, S. Ren, H. Wu, H. Li, C. Ye, K. Davey and S.-Z. Qiao, Establishing Exceptional Durability in Ultralow-Temperature Organic-Sodium Batteries via Stabilized Multiphase Conversions, J. Am. Chem. Soc., 2024, 146, 1619-1626.

29 H. J. Noh, H. Qing, P. Wang, W. Li and K. A. Mirica, Incorporating Redox-Active Hexaazatrinaphthylene into a 2D Conductive Metal–Organic Framework for Robust Sodium-Ion Batteries, Angew. Chem. Int. Ed., 2025, 65, e16381.

30 L. Wang, S. Fang, H. Wang, Q. Peng, Y. Liu, H. Dong, H. Yan, Y. Wang, S. Chou, B. Sun, Y. Xiao and S. Chen, Flexible self-supporting organic cathode with interface engineering for high-performance and wide-temperature sodium-ion batteries, Carbon Energy., 2024, 6, e632.

31 Y. Quan, W. Deng, Q. Ma, C. Guo and Z. Jin, Intermolecular Stabilization Enables Long-Life, Low-Temperature-Resilient Amino-Anthraquinone Anodes for Sodium-Ion Batteries, Nano Letters, 2026, 26, 1019-1026.

32 C. Hu, Y. Li, D. Wang, C. Wu, F. Chen, L. Zhang, F. Wan, W. Hua, Y. Sun, B. Zhong, Z. Wu and X. Guo, Improving Low-temperature Performance and Stability of Na2Ti6O13 Anodes by the Ti−O Spring Effect through Nb-doping, Angew. Chem. Int. Ed., 2023, 62, e202312310.

33 S. Xu, K. Yao, D. Yang, D. Chen, C. Lin, C. Liu, H. Wu, J. Zeng, L. Liu, Y. Zheng and X. Rui, Interfacial Engineering of Na3V2(PO4)2O2F Cathode for Low-Temperature (−40 °C) Sodium-Ion Batteries, ACS Appl. Mater. Interfaces., 2023, 15, 14329.

34 S. Zhong, Y. Yu, Y. Yang, Y. Yao, L. Wang, S. He, Y. Yang, L. Liu, W. Sun, Y. Feng, H. Pan, X. Rui and Y. Yu, Molecular Engineering on Solvation Structure of Carbonate Electrolyte toward Durable Sodium Metal Battery at −40 °C, Angew. Chem. Int. Ed., 2023, 62, e202301169.

35 B. Peng, Z. Zhou, J. Xu, N. Ahmad, S. Zeng, M. Cheng, L. Ma, Y. Li and G. Zhang, Crystal Facet Design in Layered Oxide Cathode Enables Low-Temperature Sodium-Ion Batteries, ACS Materials Letters, 2023, 5, 2233-2242.

36 Z. Y. Gu, J. Z. Guo, Z. H. Sun, X. X. Zhao, X. T. Wang, H. J. Liang, B. Zhao, W. H. Li, X. M. Pan and X. L. Wu, Aliovalent-Ion-Induced Lattice Regulation Based on Charge Balance Theory: Advanced Fluorophosphate Cathode for Sodium-Ion Full Batteries, Small, 2021, 17, 2102010.

37 Q. Shi, R. Qi, X. Feng, J. Wang, Y. Li, Z. Yao, X. Wang, Q. Li, X. Lu, J. Zhang and Y. Zhao, Niobium-doped layered cathode material for high-power and low-temperature sodium-ion batteries, Nat. Commun., 2022, 13, 3205.

38 W. Meng, Z. Dang, D. Li and L. Jiang, Long-Cycle-Life Sodium-Ion Battery Fabrication via a Unique Chemical Bonding Interface Mechanism, Adv. Mater., 2023, 35, 2301376.
